# A Hybrid Effectiveness-Implementation Trial of the ‘Power To Prevent Diabetes Program’ in Bamako, Mali

**DOI:** 10.1007/s43477-025-00199-x

**Published:** 2026-01-08

**Authors:** Sally E. Findley, Lancina Doumbia, Hamidou Oumar Ba, Bonkana Maiga, Rokiatou Koné Béréthé, Hadja Madiè Sangaré, Stéphane Bésançon, Drissa Coumaré, Lassina Coulibaly, Hamane Cisse, Salamata Y. Sidibe, Karamoko Diallo, Racky M’Baye, Mana Simaga, Moussa Coumaré, Aly Goita, Bourama Diarra, Maimouna Kanté, Fanta Sanogo, Yeya dit Sadio Sarro, Lehbib Boukenem, Sow Djeneba Sylla, Ibrahim Nientao, Hamadoun Sangho, S. Patrick Kachur, Seydou Doumbia

**Affiliations:** 1 Columbia University, New York, USA; 2 Université des Sciences, des Techniques et des Technologies de Bamako, Bamako, Mali; 3 University Teaching Hospital Gabriel Touré, Bamako, Mali; 4 NGO Santé Diabète, Bamako, Mali; 5 Centre de Santé Communautaire de Lafiabougou B5, Bamako, Mali; 6 Centre de Santé Communautaire de Doumanzana, Bamako, Mali; 7 Centre de Santé Communautaire de Banconi, Bamako, Mali; 8 Centre de Santé Communautaire de Djicoroni Para-Djenekabougou, Bamako, Mali; 9 Centre de Santé Communautaire de Sikoroni-Sourakabougou, Bamako, Mali; 10 Centre de Santé Communautaire de Lafiabougou II, Bamako, Mali; 11 National Diabetes Control Center, Bamako, Mali; 12 University Teaching Hospital of Mali, Bamako, Mali

**Keywords:** DPP adaptation, Type 2 diabetes, Diabetes in africa, Diabetes prevention and management, PPD-Mali

## Abstract

Community-based diabetes prevention and control programs are effective but limited in West Africa. We assess adoption and effectiveness of a contextually adapted Diabetes Prevention Program “Power to Prevent” (DPP-P2P) in Bamako, Mali. Guided by implementation science principles, we adapted the DPP-P2P to align with Malian culture, integrating dietary and exercise modifications. Local audiovisuals and photo guides were created for healthy food preparation and exercises. Six community health centers with high diabetes rates were randomly assigned to intervention or comparison, and 429 individuals (≥ 25 years old with diabetes, hypertension, or obesity) consented to participate (intervention = 282; control = 147). Diabetes peer educators facilitated 14 bi-weekly sessions over six months. We assessed Hemoglobin A1c (HbA1c) and blood pressure and surveyed 15 key healthy lifestyle behaviors pre-and post-intervention. Retention was 77.1%, and groups especially liked the role plays, African audio-visuals, peer educator success stories, and demonstrations. In the intervention group, individuals wanting to weigh less increased from 31.5 to 58.9% (*p* < 0.001), exercise days rose from 3.5 to 5.6 (*p* < 0.001), and the desire for a healthier diet rose by 0.64 points. Average HbA1c in the intervention group dropped from 6.5% to 5.4% (*p* = 0.001). The intervention increased the odds of lowering blood glucose below 6.5% by 2.31 times (*p* = 0.020) and blood pressure below the hypertension threshold by 2.67 times (*p* = 0.060). The Malian-adapted DPP-P2P was well accepted by the community, increasing the adoption of healthy behaviors and reducing blood glucose and hypertension. This program could benefit Mali and Sahelian countries in tackling diabetes and hypertension.

**Trial Registration:**

ClinicalTrials.gov #NCT05260879. Registered 6 December 2021—Retrospectively registered (registration link: https://clinicaltrials.gov/study/NCT05260879).

Amid the global rise in diabetes, Sub-Saharan Africa (SSA) is projected to see an increase in diabetes prevalence from 4.5% in 2021 to 5.2% by 2045 (Sun et al., 2022). Mali is one of the SSA countries where diabetes and its underlying risk factor, hypertension, have risen in recent years, particularly in urban and peri-urban areas of Bamako (Bâ et al., 2018; Organisation Mondiale de la Santé & Ministère de la Santé, 2008), as well as in neighboring rural areas (Diawara et al., 2023). A longitudinal study across four SSA countries revealed that diabetes incidence was highest among individuals with elevated blood glucose, male gender, a family history of type 2 Diabetes (T2D), hypertension, and being overweight or obese (Chikwati et al., 2025). In several high-income countries, lifestyle health promotion programs have successfully helped individuals adopt recommended behavioral changes to reduce their risk of diabetes and its associated co-morbidity of hypertension. Despite the growing diabetes epidemic in SSA, comprehensive lifestyle change programs that address diabetes have been limited in this region (Ebireri et al., 2016; Kalkonde et al., 2018; Tawa et al., 2020).

Programs developed for high-income countries often reflect their cultural contexts, making them unsuitable for low- or middle-income settings. For comprehensive lifestyle change programs to be effective in Africa, substantial cultural adaptations must be made while maintaining fidelity to elements that ensure positive outcomes (Haregu et al., 2020). To date, relatively few studies have documented attempts to adapt healthy diet or exercise recommendations to the African context. Most of these studies have focused on improving the management of diabetes or hypertension, rather than their prevention (Thuita et al., 2020; Ndejjo et al., 2022; Debussche et al., 2018; Yan et al., 2014). Additionally, they have not adequately integrated both dietary and exercise recommendations, which are crucial for preventing and managing diabetes (Muna, 2013). One exception is the South African adaptation of the Centers for Disease Control and Prevention (CDC) Diabetes Prevention Program-Power to Prevent (DPP-P2P), which maintained high fidelity to the original DPP-P2P and achieved significant reductions in glycated hemoglobin, but not in weight or blood pressure (Catley et al., 2019, 2020). More recently, an adaptation of the Finnish Diabetes Prevention Program for the African setting demonstrated that group sessions led by community health workers (CHWs) enhanced diabetes management when added to standard clinical care (Guwatudde et al., 2022). Building on this, an ongoing study in South Africa is evaluating the effectiveness of group sessions combined with text messages for diabetes prevention (Hill et al., 2023a, b). Considering the notable cultural, contextual, and linguistic differences between Mali and South Africa, this study aimed to assess the potential acceptability and effectiveness of an adapted version of the DPP-P2P in urban and peri-urban areas of Bamako, Mali (Doumbia et al., 2024). In line with other lifestyle change initiatives, our adaptation highlights the importance of addressing hypertension, as demonstrated in recent adaptations of the DPP, which also tracked changes in hypertension (Devaraj et al., 2021; Kramer et al., 2009). Moreover, a longitudinal study of DPP outcomes showed that the lifestyle change program improved diabetes control, as well as reducing cholesterol and blood pressure levels (Diabetes Prevention Program Outcomes Study Research Group et al., 2013). Consequently, recognizing the overlap in lifestyle change messaging for diabetes prevention and management, our study will address both prevention and improved control of diabetes (Hill et al., 2023; Hill et al., 2023a, b).

## Methods

### Study Design

This is a type II hybrid-effectiveness implementation design to adapt and assess the DPP-P2P. Qualitative feedback and self-reported behavior change assessments first were utilized in the development of the Malian DPP-P2P adaptation. This included introducing video materials and culturally tailored guides for food preparation and exercise. Implementation research continued throughout the effectiveness trial, a cluster-randomized study with a controlled before-and-after design, comparing differences within and between intervention and comparison groups. We evaluated changes from pre- to post-participation for key outcome measures. The study protocol was approved by the ethics committee of the Faculty of Medicine and Odonto-Stomatology of Bamako (#2020/207/CE/FMOS/FAPH) and is registered with ClinicalTrials.gov (#NCT05260879). The study is reported in accordance with the Consolidated Standards of Reporting Trials (CONSORT) checklist (Supplementary file 1).

### Study Sites and Population

**Figure 1:**
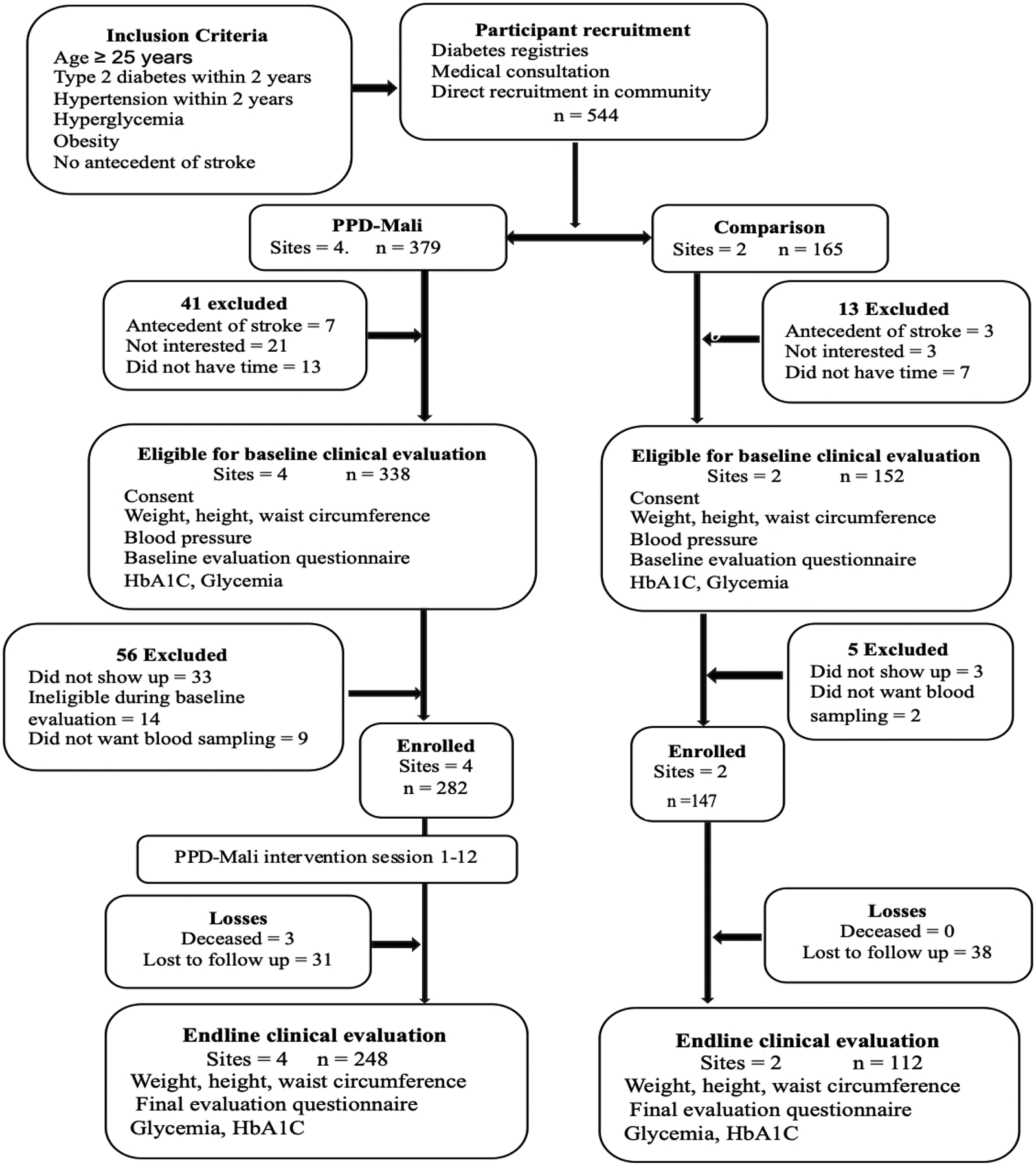
Participant Flow Diagram *Note*. Figure 1 describes the inclusion criteria and participants’ recruitment process. A total of 544 patients were recruited and allocated into the Intervention group, the Mali Diabetes Prevention Program (PPD-Mali), and the Comparison (No intervention). In the PPD-Mali group, 282 patients were enrolled, of whom 248 (88.0%) completed the study. In the comparison, 147 patients were enrolled, of whom 112 (76.2%) completed the study. Weight, height, and waist circumference were measured at baseline and endline.

We used the combined data from all six Bamako health districts to identify the two health districts with the highest number of diabetes patients diagnosed in the previous two years. Within each selected district, the community health centers (CSCOMs) were ranked in descending order by their total patient population. A selection interval was set at one-third of the district’s total case numbers, and with a random start, three CSCOMs were randomly selected in each district. These six CSCOMs were randomly assigned to one of three study groups: couples, individuals, or a comparison group, with two CSCOMs per study group. Medical officers at each CSCOM compiled a list of eligible individuals living in their catchment service area: those aged 25 and over, diagnosed with type 2 diabetes or hypertension within the past two years, or overweight and considered at risk for either disease, as identified in diabetes registries, or seen at routine consultations or during home visits by CHWs. Of the 544 eligible individuals initially deemed eligible, those who remained eligible after baseline assessments and agreed to participate provided written informed consent. Individuals with type 1 diabetes or gestational diabetes were excluded. At the two Couples sites, participants (*n* = 148) were initially enrolled as individuals. After the second session, they were invited to bring their spouse or partner to enroll in the study. Because only 35% (*n* = 52) of partners chose to participate in the Couples group, we do not include them in the analysis and treat the participants initially enrolled in the Couples sessions as individual participants. Figure 1 displays the participant flow diagram. Recruitment occurred from November 26, 2021, to December 24, 2021.

### Intervention

The PPD-Mali intervention consisted of 14 educational sessions adapted from the CDC’s DPP-P2P (National Diabetes Education Program, 2006). Drawing from the twenty-year review of the National Diabetes Education and Prevention Program (Siminerio et al., 2018), we identified key elements for implementing the Power to Prevent program. The modifications necessary for implementation in the Malian context are highlighted in bold in Table 1.

While we preserved the core content and approach of the DPP-P2P, several key changes were essential for the program’s effective delivery with high fidelity. First, we adjusted the presentation style to suit the anticipated low literacy level of participants, converting the DPP-P2P session guidelines and flipcharts into French slide presentations. All presentations were enhanced with culturally relevant images and storylines, showcasing African participants eating local foods and exercising in typical urban African settings, preferably captured by the study’s videographer in Bamako neighborhoods. The PEs, fluent in French and Bambara, were trained to describe the slides in Bambara, using guidelines for verbal translation provided by the study coordinators during the training. Healthy eating recommendations were adapted to the food purchasing and consumption patterns of Bamako, where there is little consumption of packaged foods. Recognizing that most individuals eat together as a family from a common bowl, participants making changes to sauce preparation would need to gain the support of the male head of household and other adult women sharing cooking responsibilities. Therefore, sessions were added on how to gain the support of others in the household. The study’s videographer collaborated with the research team to create videos of CHWs purchasing and preparing food using the healthy sauce guidelines, some of which were featured in the pictorial cookbook. We replaced the sessions on label reading and calorie counting with measurements of portion size, emphasizing the reduction of portions of rice and other starchy staples. In Bamako, residential areas lack sidewalks or parks, so exercise recommendations were adapted to at-home workout routines or neighborhood dance and exercise groups organized through the program. The videographer also prepared videos and a photo guide of the recommended at-home exercises (See supplemental material for the pictorial cookbook and exercise guides). Finally, in addition to the original focus of the DPP-P2P on diabetes prevention among at-risk individuals, our adaptation highlighted how these recommendations could also enhance diabetes management.

To achieve these changes, we employed an iterative, participatory approach to adapt the 12 DPP-P2P sessions for use in Bamako (Minkler & Wallerstein, 2003; Siminerio et al., 2018) by: (1) Initially translating the 12 DPP-P2P sessions into 12 DPP-Bamako sessions by the multidisciplinary research team; (2) Gathering feedback from stake-holders at the ministry, organizational partners, and medical care providers; (3) Conducting focus groups with CHWs; (4) Further tailoring to the group presentations, including subdividing two sessions to focus their content better; (5) Running a pilot study with 45 participants diagnosed with or at risk for T2D to obtain feedback on the adaptation; (6) Finalizing the adaptation of the DPP-P2P for use in Bamako (Doumbia et al., 2024). The completed adaptation was a six-month intervention package consisting of fourteen three-hour PPD-Mali sessions, delivered by a trained PE and facilitated by a CHW (see Table 2).

At each intervention site, participants were divided into five groups of 10 to 15 individuals based on their neighborhood proximity, with each subgroup attending on their designated day of the week. Sessions combined didactic portions, presented by the PE, with group discussions, activities, and role-plays, facilitated by the CHW. These activities were designed to build the confidence of participants, particularly women, in promoting healthy eating and lifestyles to their husbands and other household members. Similar to the original DPP-P2P sessions, the PPD-Mali sessions began with participants sharing their successes and challenges in implementing recommendations from the previous session. Feedback assessments employed the stages of change approach (Prochaska & DiClemente, 1983), asking participants about their actual adoption of the 15 recommended behaviors in the past week and their plans to adopt any of these behaviors in the coming weeks. Between sessions, each CHW visited their assigned group members at home to support their planned activities. They also encouraged those who missed a session to attend a make-up session with another group at their CSCOM, reminding them of the next session date. The intervention was implemented between November 26, 2021, and June 28, 2022. Individuals in the comparison group only participated in the program when completing the baseline and endline assessments. Among them, those with diabetes continued with usual care, which included monthly visits to the district referral center for a clinician to review their care. This may have involved diabetes control medications (Glibenclamide, Metformin, or insulin) and blood glucose monitoring.

### Data Collection Procedures and Tools

Qualitative feedback was gathered during the cultural adaptation of the DPP-P2P. Effectiveness was assessed through both surveys and clinical evaluations. The CSCOM medical officers conducted clinical assessments at the beginning of session 1 (baseline) and after session 12 (endline). They recorded participants’ age, height, weight, abdominal circumference, pulse rate, and blood pressure (BP). They also documented prior diagnoses of diabetes or hypertension, along with details of current treatments, including oral anti-hypertensives, hypoglycemic drugs, or injectable insulin treatments. After participants had fasted for 8 h from the night before, blood samples were collected in serum separator tubes (SST) and ethylenediaminetetraacetic acid (EDTA) tubes, which were sent to the university laboratory for glycemic and Hemoglobin A1C (HbA1c) tests (Pentra C400, Irvine, CA, USA). For extremely high values, samples were diluted and reanalyzed to ensure accurate interpretation. HbA1c results were classified as without diabetes (< 6.5%) or diabetes (≥ 6.5%) (The International Expert Committee, 2009). Clinical hypertension was defined as systolic blood pressure (SBP) ≥ 140 mmHg or diastolic blood pressure (DBP) ≥ 90 mmHg (World Health Organization, 2021), using standard definitions for both conditions.

Our assessments, completed at baseline, midline after the fourth session, and endpoint, were designed to track progress through the pre-contemplation, contemplation, preparation, action, and maintenance stages of change of the Transtheoretical Model, as initially developed by Prochaska and DiClemente (Prochaska & DiClemente, 1983). Participants were asked about their knowledge of diabetes and its control, as well as their plans and actual adoption of the 15 key healthy eating and activity recommendations included in the PPD-Mali adaptation of the DPP-P2P (Doumbia et al., 2024). Facilitators guided participants through the questions, translating them into Bambara, and recorded their responses on the participants’ survey form. After the “Move More at Home” sessions (sessions 4 A and 4B), participants received Pedometers (Pedusa PE-771, PedometersUSA) to track their daily steps for weekly reports. Following the “Healthy Eating” sessions (sessions 5 A and 5B), participants used a pictorial food consumption checklist to track their portions in each major food category in the week prior to the session (See supplemental material for the pictorial food log). The midline assessment was used to evaluate variations in the uptake of behavioral changes by site, enabling the researchers to address inconsistencies in session delivery across sites. A shorter feedback survey was administered after each session to assess whether participants had adopted any of their chosen goals for specific behavior changes. These feedback surveys were used to determine participants’ satisfaction with the sessions and to guide the training and re-training of the facilitators. For each clinical evaluation and individual session, standardized scannable paper data collection forms were developed in French using Remark Office Optical Mark Recognition (OMR) software (Gravic, Inc; Malvern, PA, USA). Completed forms were scanned with equipment connected to the Remark OMR software. Study team members reviewed the scanned data for incomplete or anomalous entries and resolved these by consulting the original data collection sheets.

### Training of Key Personnel

The PPD-Mali intervention was implemented using a team approach, training and coordinating implementation through key individuals at each site. This included the medical officer, two laboratory technicians, six community health workers (CHWs), and four diabetes peer educators (PEs) from the Mali National Diabetes Association. Four study site coordinators oversaw the activities from training through session implementation. The medical officers at each site were trained to identify eligible individuals and to conduct the clinical evaluations. Lab technicians were trained in standardized protocols for collecting and packaging blood samples for transport to the university laboratory. Four Peer Educators (PE) were recruited from the Bamako diabetes treatment center, where they had demonstrated effective diabetes management and received training to serve in this role. Each PE was assigned to an intervention site, where they led the delivery of the sessions. Prior to implementation, the PEs were trained to use the presentation materials and deliver the session’s contents in Bambara, the lingua franca of Bamako. Utilizing trained and experienced PEs was a key strategy to ensure high fidelity to the adapted sessions and transfer skills to CHWs and participants. CHWs were trained to encourage participation, lead role-plays, and conduct demonstrations in the sessions, and to counsel them on implementing dietary and exercise recommendations during home visits to participants. At each site, implementation was monitored by one of the four medical interns working with the study, who also coordinated the research activities there. The study was paused after the sixth session for the Muslim fasting month, when a daytime fast is widely observed in Bamako. The PEs and CHWs were retrained on the remaining sessions before resuming the study with session 7.

### Implementation Science Considerations

Adapting evidence-based interventions to contexts that are significantly different from those in which they have previously proven effective is a crucial concern for implementation scientists (Escoffery et al., 2019). This is particularly important for ensuring that adaptations remain fidelity-consistent (Powell et al., 2015). We utilized the four primary domains in the Exploration, Adoption/Preparation, Implementation, and Sustainment (EPIS) Framework (Aarons et al., 2011) to identify and ensure consistency among several strategies in our adaptation. As highlighted in Table 1, a key Innovation Factor in DPP-P2P is task shifting responsibility for lifestyle change from clinical providers to other team members. In adapting PPD-Mali, the sessions employed a team approach where the PE’s didactic presentations on healthy eating and physical activity were complemented by the activities facilitated by the CHWs, who led the group discussions, role plays, demonstrations, and videos to engage participants in active learning. Feedback from the participants consistently highlighted their appreciation for learning from the PEs about managing their diabetes, while the role plays and demonstrations led by the CHWs were repeatedly the most appreciated parts of each session. A major innovation was the introduction of videos demonstrating how to prepare healthy sauces, accompanied by pictorial guides for the healthy preparation of the five main sauces that comprise most families’ diet. We created an exercise regimen suitable for home practice and provided videos and pictorial guides for these exercises. We developed interactive and supportive training and supervision to support these cadres, ensuring they could deliver each session as designed. Student interns observed each session and signaled to supervisors when additional coaching was needed. Among the strategies that addressed the Inner Context was deploying CHWs to encourage enrollment of potential participants. We devised a clinical tracking data collection system to promote continuity of care between clinical providers and CHW/PE teams implementing the PPD-Mali sessions. We also recognized that participants would need to take action based on PPD-Mali within the Outer Contexts of their homes and communities. Strategies to address this included specific training on skills for discussing changes with spouses and other household members. CHWs encouraged participants to share their progress and planned next steps toward achieving their lifestyle goals. Feedback from participants in the last few sessions showed that they highly valued this collective discussion of their successes and challenges.

### Study Outcomes and Measures

The primary effectiveness outcomes were the adoption of the 15 specific healthy eating or exercise behaviors recommended by the program. We employed the Prochaska model approach to reasoned behavior change, evaluating participants’ intentions to adopt each of the 15 recommended changes and the steps taken toward those changes (Prochaska & DiClemente, 1983). Participants self-assessed their behavior change before each session as part of the feedback discussion. Starting after session 4B, participants recorded their exercise minutes and daily steps from the previous week in preparation for the next session, using pedometers for tracking. After Session 5B, participants used a pictorial food log to track the number of portions of key foods they consumed in the week preceding their next session. Our focus was on changes in the portions of rice consumed. Beginning with session 6, facilitators also compiled data from these logs onto the data collection forms. The baseline, midline, and endline assessments included behavior change questions, food consumption records, physical activity records before the session, and knowledge of diabetes and its prevention. The secondary effectiveness outcomes were reduction in diabetes status, as measured by HbA1c ≥ = 6.5% (The International Expert Committee, 2009), and hypertension (SBP ≥ 140 mmHg or DBP ≥ 90 mmHg) (World Health Organization, 2021). Obesity was assessed using body mass index (BMI), from which we developed a categorical indicator of normal (BMI < 25 kg/m^2^), overweight (BMI ≥ 25 kg/m^2^ & <30 kg/m^2^), or obese (BMI ≥ 30 kg/m^2^) (American Diabetes Association Professional Practice Committee, 2023). We also asked if the participant had ever been told by a health care provider that they had type 2 diabetes or hypertension.

### Data Analysis

We report implementation outcomes and knowledge gained at the conclusion of the final PPD-Mali session, using simple frequencies among study participants without performing intergroup statistical comparisons. The Chi-Square test was used to assess distributional differences in behavioral outcomes between the two study groups. A two-sample t-test was employed to evaluate differences in effectiveness outcomes between the comparison and intervention groups at baseline. Due to the limited number of enrolled spouses or partners in the study (52 out of the expected 150), we were unable to perform separate, detailed contrasts between the Individual and Couples groups. Consequently, all subsequent analyses contrast the comparison and the intervention, including the Individual groups and only the originally recruited individuals of the Couples group.

The difference-in-differences method was employed to determine whether behavioral or clinical outcomes during the 6-month intervention period were more favorable for the intervention groups compared to the comparison group. Ordinary Least Squares (OLS) regression was used to estimate the contribution of the intervention to changes in exercise days and of desire for a healthier diet, controlling for the baseline characteristics of age, gender, overweight status, and prior diabetes diagnosis. We used logistic regression to estimate the contribution of intervention participation to the odds of achieving diabetes and hypertension control, while controlling for exercise days and the same risk factors as above. For the hypertension control, estimations prior hypertension diagnosis was included instead of prior diabetes diagnosis. All analyses were conducted using Stata version 15.2.

## Results

As shown in Table 3, there were no significant baseline differences between the comparison and intervention groups. Participants’ mean age was 54, with 68% being female. A third (75%) of the participants were overweight or obese, with an average BMI of 28.9 kg/m^2^. Just over half (57.9%) had a prior diagnosis of diabetes, and the average HbA1c level was 6.8%. Only 12.5% were on insulin. Two-thirds (68.6%) had been diagnosed with hypertension, with a mean systolic pressure of 150.8 mmHg and diastolic pressure of 91.9 mmHg. Half reported being on hypertensive medications.

**Figure 2:**
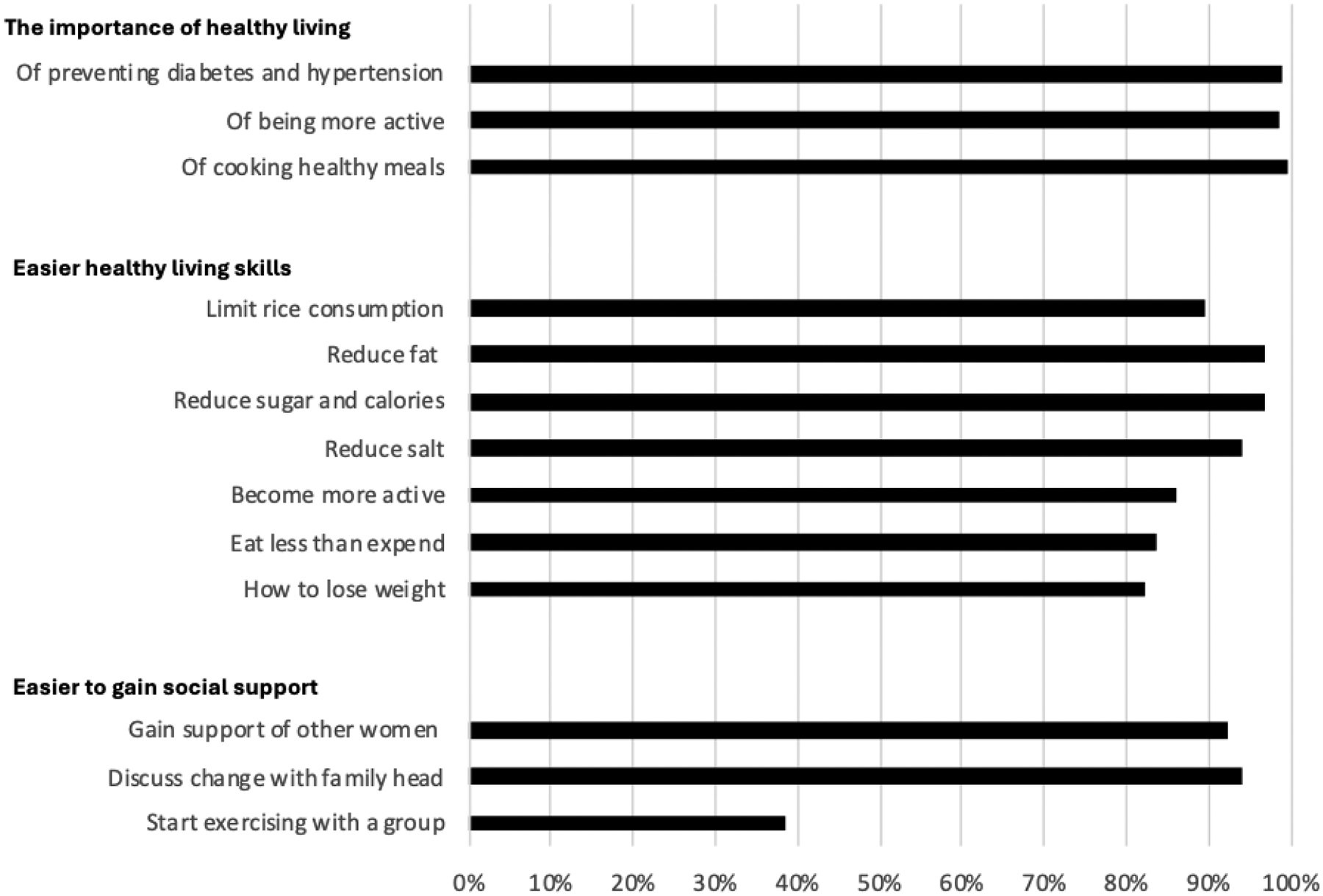
What Participants Learned from the Program

Implementation outcomes indicate high uptake and recall of key program messages, with over 80% of participants accurately recalling all three messages promoting healthy living and all seven messages focused on healthy living skills at the endline (See Fig. 2). More than 90% of participants recalled learning how to gain support from other women and the family head.

Individuals participating in the intervention experienced significant changes in several of their goals for staying healthy (See Table 4). For instance, while the comparison group did not report changes in their general health perception, the intervention group saw an increase in those who believed themselves to be in “good” to “very good” health, rising from 34% to 72% (Chi-Square = 6.51, *p* = 0.011). In the intervention group, the percentage wanting to weigh less increased from 32% to 59% (Chi-square = 24.8, *p* < 0.001), contrasting with declines in the comparison group. The second panel of Table 4 shows that the number of days participants exercised nearly doubled among the intervention participants, increasing from 3.5 to 5.6 days per week (*t*-test = 8.03, *p* < 0.001), while exercise days remained unchanged in the comparison group. In both groups, the reported days of eating out decreased to once or fewer times per week.

There were significant improvements in the clinical markers for diabetes and hypertension (Table 4). In the intervention group, HbA1c declined from 6.52% to 5.35% (*t*-test = 3.35, *p* = 0.001), while the comparison group showed no changes. Those classified with diabetes rose from 40% to 60% in the comparison group, whereas the intervention group saw this figure halve, dropping from 56% to 22% (*t*-test = 5.72, *p* < 0.001). BMI did not change significantly in either group, but both groups experienced a significant decline in systolic blood pressure, from approximately 150 to 143. Diastolic blood pressure declined significantly in the intervention group, from 92.2 to 89.9 (*t*-test = 2.22, *p* = 0.100), while it remained unchanged in the control group. Those classified as hypertensive declined in both groups, from around 40% to 28–30% (*p* < 0.028).

**Figure 3:**
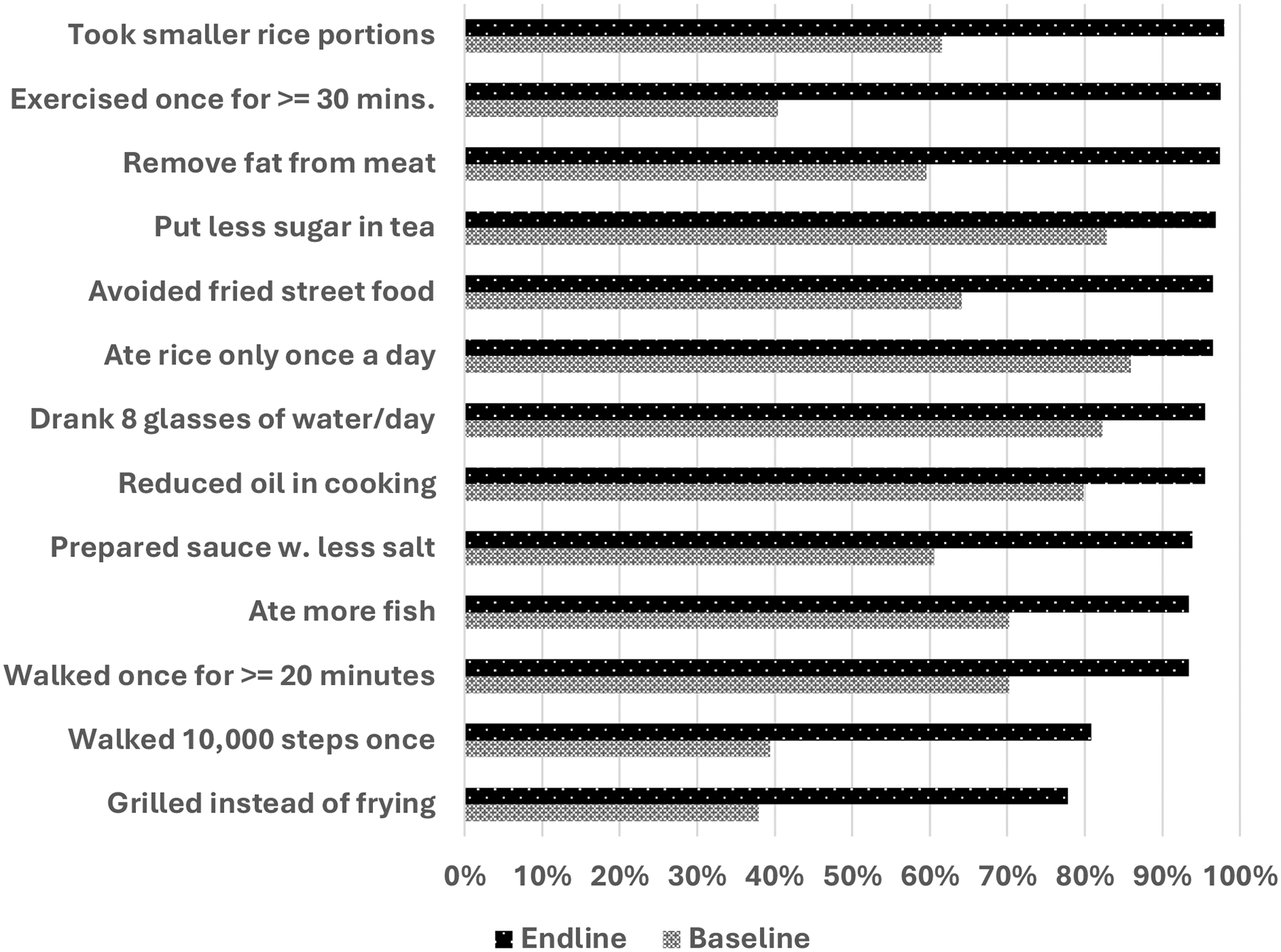
Baseline to Endline Changes in Adoption of Recommended Healthy Behaviors among Intervention Group Participants (n=237) *Note*. All differences significant at the .01 level or higher, per paired t-test

In the intervention group, we monitored over a dozen healthy behaviors that participants could adopt between sessions. At baseline, the percentage of participants engaging in any of these behaviors increased from 38% to 86% to 78% to 98% at endline (See Fig. 3). At the end of the intervention, over 90% had adopted 11 of the 13 recommended healthy behavior changes.

Finally, we assessed the association between participation in the intervention and the primary behavioral and secondary clinical outcomes, controlling for the confounders of age, gender, prior diagnoses, weight status, and changes in exercise levels—the behavior change most closely linked to the intervention. The OLS coefficients indicate that participation in the intervention increased by 1.8 the number of weekly exercise days of more than 20 min (See Table 5), and participation increased the number of individuals who strongly desired a healthier diet. Logistic regressions reveal that when controlling for confounders (e.g. diabetes diagnosis at baseline), participation in the intervention increased the odds of achieving an HbA1c below the diabetes threshold of 6.5% by 2.31 (95% CI 1.14–4.65, *p* = 0.02). However, for women, as compared to men, the odds of this reduction were reduced by 0.48 (95% CI 0.22–1.04, *p* = 0.06). Increasing exercise days did not further impact achieving diabetes control after adjusting for intervention participation. Finally, after controlling for the confounders (e.g., hypertension diagnosis at baseline), the intervention increased the odds of reducing hypertension below the threshold of SPB > = 140 mmHg or DBP > = 90 mmHg by 2.67, compared to those in the comparison group (95%CI 0.97–7.35, *p* = 0.06). Overweight or obese individuals had a 2.85 higher odds of reducing their hypertension (95% CI 0.88–9.23, *p* = 0.08). In the HbA1c regression analysis, increased exercise days did not significantly affect the odds of reduced hypertension, after adjusting for intervention participation.

## Discussion

The PPD-Mali study evaluated the potential effectiveness of an adapted version of the DPP-P2P, specifically tailored for Francophone West Africa and the context of low-income neighborhoods in Bamako, Mali’s capital. We adhered to the features associated with the positive outcomes of the DPP-P2P, namely delivering to small community-based groups by CHWs who facilitated group interactions, using simple graphic materials to convey essential concepts, and encouraging participants to gradually change their eating and exercise habits through small steps taken from session to session. Our adaptation was not only language and culturally appropriate for the Bamako communities, but it also included extensive use of role plays, demonstrations, cooking and exercise videos, and pictorial guides, with additional facilitation by PEs who had successfully controlled their diabetes. The implementation outcomes found that nearly all participants could recall key content pertaining to the importance of healthy living, healthy living skills, and social support strategies. Along with the high satisfaction expressed for the program and its CHW and PE facilitators, the program’s 77% retention rate through the end of the program attests to its popularity and acceptability. Participation in the PPD-Mali sessions exceeds the average attendance rate of 61% observed in sessions in the South Africa DPP study (Catley et al., 2022). Our attendance level compares favorably with those observed in the US DPP, where fewer than half of the study participants attended all sessions (Ely et al., 2017).

Given the limited availability of healthy lifestyle programs in Bamako for individuals diagnosed with diabetes, and the recent shift in diabetes lifestyle programs to include participation from those at risk or already diagnosed (Wang & Abbott, 1998; Diabetes Outcomes Study Research Group, 2013; Devaraj, 2021; Guwatudde, 2022, Hill, 2023), we included both those at risk and those already diagnosed with diabetes, the latter constituting over half (58%) of our study. They responded positively to the PE’s stories about how they managed their diabetes. Although we did not specifically inquire about the mix of diagnosed and at-risk participants, it is likely that those at risk recognized their status as “not yet” affected. By participating in the groups, they learned steps to prevent diabetes, rather than perhaps hoping it would not affect them.

The majority of our study participants were women. Since study recruitment was based at the CSCOM, this aligns with the gender differences in CSCOM consultation rates, where women are more frequent visitors than men. A similar low participation rate among men has been reported in other diabetes lifestyle interventions (Catley et al., 2022; Knowler et al., 2002; Mathews et al., 2018; Guwatudde et al., 2022). The lower availability of men was also shown in the under-recruitment for the Couples study group, for which only one-third of the initial participants successfully recruited their partner. However, the female majority is likely an advantage in terms of implementing healthy diet recommendations. Our program recognized the key role of women in Malian culture, where women in households share the responsibilities of purchasing and cooking meals for their families. Thus, by teaching the participating women how to adopt a healthier diet, our program was likely to spread the recommended cooking practices beyond the individual participants to other women in the household, leading to further dietary changes among all household members.

At the end of the study, nearly three-fourths (72%) of the intervention participants reported being in good to excellent health. More participants in the intervention group expressed a desire to exercise more and eat a healthier diet. In contrast, the comparison group showed no change in self-assessed health status or lifestyle goals. While changes in self-perception do not always align with shifts in behavior, they did in the intervention group. Figure 2 illustrates how the intervention group transformed their goals into action, as evidenced by increased exercise, the use of recommended healthy cooking practices, and a reduction in the consumption of rice and fried foods. This may reflect the interactive aspect of our adaptation of the DPP-P2P intervention, where each session provided an opportunity for participants to share their experiences on overcoming barriers and difficulties in adopting the recommended behaviors. The group participation effect was also identified in the DPP (Siminerio et al., 2018). Social support from other participants may have contributed to the significant increase in exercise days among the intervention participants, particularly if they started walking with others in the group. These changes in behavior likely contributed to their increased sense of good health, a pattern found by others who have demonstrated a positive relation between levels of physical exercise and quality of life (Esteban y Peña et al., 2010).

In the intervention group, the number of reported exercise days increased to 5.6 days per week, representing a 59% increase, whereas the comparison group showed no change. After controlling for their gender, age, overweight status, and diabetes diagnosis, participants in the PPD-Mali program increased their weekly exercise days by 1.83 days. Although increasing moderate-to-vigorous exercise has long been identified as a way to lower the risk of cardiovascular diseases, there are only a handful of studies assessing exercise promotion programs for persons with diabetes in Africa (Aune et al., 2015; Rao et al., 2022; Tuomilehto et al., 2011). The increase in physical activity levels in our intervention group exceeds that observed in exercise promotion programs for patients with diabetes in Ethiopia and Reunion, but parallels those observed in a similar study in Iran (Debussche et al., 2012; Eshete et al., 2023; Rouholamini et al., 2020). One factor that may have contributed to the success in increasing exercise days was our awareness that the neighborhoods lacked safe and accessible outdoor exercise options. This led us to develop a series of exercises to be performed at home, which we taught in the group sessions and supplemented with a pictorial guide.

Compared to the comparison group, participation in the intervention significantly shifted participants toward adopting healthier diets, after adjusting for baseline characteristics of gender, age, weight status, and diabetes status. Our findings of both desire for and adoption of recommended dietary practices contrast with the low level of uptake of dietary recommendations among persons with diabetes in Ethiopia and Nigeria (Okunlola et al., 2022; Worku et al., 2015). Despite budget and availability constraints potentially limiting access to healthy diets in African cities (Tacoli et al., 2024), our intervention’s demonstrations and pictorial guide to cooking healthy sauces enabled the study participants to shift toward healthier diets.

The six months during which participants attended the PPD-Mali sessions appear to have been sufficient for their behavioral changes to align with improvements in their clinical outcomes. While the HbA1c level increased slightly for the comparison group, it declined by 18% to an average of 5.35% in the intervention group. After controlling for baseline characteristics and prior diabetes diagnosis, those in the intervention group had 2.31 times greater odds of achieving an HbA1c level indicating control of their blood glucose level, compared to the comparison group. Compared to men, these higher odds were partially offset for women by a slight reduction of 0.48 in their odds of diabetes control. Our findings are comparable to those found in a parallel adaptation of the DPP in South Africa, which also documented significant declines in HbA1c among intervention participants (Catley et al., 2022). Both the South African study and our study suggest that part of the positive outcomes may be due to the DPP-P2P program style, featuring CHW-led group sessions distinct from diabetes clinical care. This separation from diabetes clinical care may contribute to the more positive outcomes than in parallel studies in Uganda and South Africa, where group lifestyle change sessions were integrated into facility care for individuals at risk of or already diagnosed with diabetes, but found no additional improvements in hyperglycemic control after controlling for similar baseline characteristics as in our analyses (Guwatudde et al., 2022). Although we found no independent effect of exercise days on the odds of blood glucose control, our overall intervention effect findings align with those of others who report associations between the changes in diet and exercise behaviors promoted by our intervention and improved HbA1c (Bore et al., 2023; Kim et al., 2013; Shah et al., 2021).

Although blood pressure reductions were not a primary outcome of the study, our intervention correlated with declines in both systolic and diastolic blood pressure. The declines in the intervention group’s diastolic pressure were significantly greater than those in the comparison group. The intervention group’s systolic pressure declined from 151 to 143, while their diastolic pressure declined from 92 to 90. After controlling for gender, age, overweight status, exercise days, and prior hypertension diagnosis, those in the intervention group had a 2.67 increase in their odds of achieving hypertension control, albeit highly variable and only marginally significant. While the South African adaptation of the DPP did not show a substantial reduction in blood pressure, our results align with those of the DPP-Group Lifestyle Balance program, which resulted in sustained reductions in systolic and diastolic blood pressure (Catley et al., 2022; Devaraj et al., 2021; Diabetes Prevention Program Outcomes Study Research Group et al., 2013; Kramer et al., 2009). While our intervention’s effect was not as strong on hypertension as on diabetes prevention and control, the positive findings for blood pressure reduction suggest the merit of integrating hypertension control into diabetes prevention programs.

Our study design combined implementation science with an effectiveness trial and had several limitations. First, the design was powered to observe outcome changes by study group, but not for a detailed analysis of the specific intervention elements contributing to outcomes within the study groups. Second, the number of partners in the Couples study group was insufficient for an appropriate comparison of enrollment between the Couples and the Individual group. In subsequent studies, we recommend recruiting Couples together at the beginning, ensuring the requisite number of dyads. Third, changes in weekly diets or exercise patterns were self-reported for both study groups. Thus, there could be a self-report bias, with differences between the two groups reflecting changes in perception rather than actual changes in their diets or exercise. Since we lacked more detailed data on the comparison group’s diets and exercise patterns, we were unable to assess detailed behavioral changes across the entire study population. Even with its pictures, the detailed pictorial daily food consumption checklist was difficult for participants. We recommend focusing on the key foods, such as rice and other high-density food items, particularly fried foods, as these targeted items appear to have been more reliably tracked. Similarly, although participants wore pedometers, there were issues with their use. If resources had permitted, it would have been preferable to use mobile phone applications and paid phone subscriptions for the duration of the study. Fourth, this study was implemented over a period of six months, which is half the time typically required for implementing DPP-P2P interventions. The shorter duration in our study may not have been sufficient to generate the behavior or weight changes that participants sought to achieve. Modifying diet and exercise behaviors is a long process, crucial for the success of diabetes management interventions. Fifth, we did not assess triglycerides and low-density lipoprotein cholesterol, which would have helped determine the short-term impact of dietary changes. We also did not include the more detailed clinical assessments to track changes in hemoglobin that could have been affected by sickle cell anemia or malaria, both of which are potentially present in our population. Finally, both the original DPP-P2P and our adaptation for PPD-Mali deliver a complex package of intervention elements and implementation strategies for lifestyle change. In this study, we focus on the impact and effectiveness of the adapted intervention as a whole. Future work may help elucidate the most essential and cost-effective elements to be retained in a scalable lifestyle intervention package.

## Conclusion

As in the original DPP-P2P, our Malian adaptation of DPPP2P facilitated greater adoption of healthy diets and active exercise. We attribute this to the extensive cultural adaptation of the DPP-P2P guidelines, with audio-visual and interactive elements suitable for both CHWs and participants. In the six-month program, the participants experienced significant declines in their blood glucose and blood pressure levels. While a follow-up study is necessary to assess the long-term effects of the intervention, the PPD-Mali program has promise for assisting Mali and other Sahelian countries with similar cultures in addressing the burden of diabetes and its associated risk factor, hypertension.

## Supplementary Material

Exercise booklet

Nutrition booklet

Weekly food log

CONSORT checklist

**Supplementary Information** The online version contains supplementary material available at https://doi.org/10.1007/s43477-025-00199-x.

## Figures and Tables

**Table 1:** Key Implementation Elements of DPP Power to Prevent Modified in the Mali PPD Adaptation

Power to Prevent Key Elements	Power to Prevent	Malian Adaptation
Context	Americans in community-based groups, e.g. YMCA	Community-based, local health clinic
Group Session leadership	CHWs or other non-health professionals, literate in English or Spanish	Diabetes peer educators, literate in French and Bambara CHWs, **speaking but not literate in Bambara**
Session Content	T2D risk and prevention Healthy Eating- balanced diet, reducing fats and calories Caloric balance Physical Activity – more sports and exercise Healthy Heart Drink fewer sugar sweetened drinks Eating out Reduce salt, smoking, alcohol, stress	T2D risk reduction & control Healthy Eating-more balance, reduce fats, starches, **& portions** **Healthy menus and how to prepare them** Physical Activity – **at-home exercise or group walking** Healthy Heart Drink more water, **less sugar in tea** **Street food to avoid** Reduce salt, alcohol, stress
Target population	Americans, including low-income and minority populations, literate in English or Spanish	Low-income, **low-literacy Africans in the city of Bamako, Mali**
Behavior change promotion strategy	CHW-led discussion groups Individualized game plan	CHW-led discussion groups Individualized game plan **Pictorial messaging** **Role plays** **Cooking and activity demos, with pictorial booklets** **Family-oriented**
Tracking adoption	Weekly game plan Calorie and activity trackers	Weekly game plan **Group discussion of successes & challenges** **Pictorial portion tracker** **Pedometers for steps** **Weekly exercise minute tracker**
Acceptability	Retention Qualitative feedback	Retention Qualitative feedback
Feasibility	Pre-tests and pilots	Pre-test and **stepwise evolution of sessions**

**Table 2:** Diabetes Prevention Program Power2Prevent Adaptations for Mali

Original DPP-P2P sessions	Malian adaptation	Major elements added or changed
1.Welcome to the DPP program	1.Welcome to PPD-Mali	Intro to DPP-P2P. Substitute “What is Diabetes? educational materials from Santé Diabète Mali, adding Bamako pictures
2. Small Steps lead to Big Rewards (SSBR)	2. Small Steps for you and your spouse	Modify flipchart story to have Malian characters Adapt SSBR game plan, food and activity trackers Add role plays and games illustrating spouses supporting each other Adapt the 50 choices to the Malian context
3.Getting started with your plan	3. Engage the family for social support	Provide guidance for gaining support of the family Adapt Benefits of Social Support module (# 10) Add role plays about engaging family head
4. Move More	4A. Move More- At home 4B. Using the exercise booklet and trackers	Importance of exercise for energy balance Add Exercise demos by Malian exercise coach Distribute and explain exercise guide booklet Distribute and demonstrate pedometer use Show how to record steps and minutes on tracker
5. Reduce your calorie intake	5A. Small steps toward healthy eating 5B. Learning to cook healthy	Introduce healthy eating concepts using adapted Benin healthy eating “hut” Introduce Mali pictorial cookbook with low-glycemic index of Malian sauces
6. Control temptation	6. Eat less but better	Adaptation of DPP session 9, with more emphasis on portion sizes instead of calories Role play to estimate portions consumed from communal pot
7. Resolve problems	7. Resolving problems	Additional role plays and discussions on handling flagging motivation to change
8. Four keys to eating out	8. Five keys to healthy eating out	Remove items pertaining to reading menus Substitute recommendations on street food and buying food outside home and during celebrations
9. More volume, fewer calories	9. Drink water, not sodas	Add a separate module on drinking water, part of DPP session 9. Messages on reducing alcohol and beer, and restricting sweetened tea and juices
10. Have a healthy heart	10. Healthy heart diet	Adaption of DPP session 10 with a pictorial introduction to hypertension Emphasize reducing salt, bouillon cubes, and sugars
11. Benefit from social support	11. How to keep your family motivated	Adaption of DPP session 12, with more emphasis on motivation of spouse. Additional discussion on gaining support from family
12. Prepare for the long term	12. Looking backward to look forward	Celebration to thank each other, spouses, family, and CHWs

**Table 3:** Characteristics of the Study Population by Intervention Group

Characteristics	Comparison group (n=106)	Intervention group (n= 237)	Total (n=343)	Two-sample t-test	*p value*
Mean age (years)	54.4	53.4	53.7	0.700	0.484
Female (%)	65.1%	69.6%	68.3%	0.847	0.397
Waist circumference (cm)	94.6	94.9	94.8	0.217	0.828
Overweight or obese	75.8%	74.3%	74.7%	0.291	0.772
BMI (kg/m^2^)	28.6	29.1	28.9	0.592	0.554
Diabetes diagnosed	52.8%	59.5%	57.6%	1.174	0.241
Hb A1c (%)	6.40	7.00	6.81	1.432	0.153
On anti-diabetes medication(%)	13.1%	12.2%	12.5%	0.250	0.803
Hypertension diagnosed (%)	72.6%	66.7%	68.6%	1.028	0.305
Systolic BP (mmHg)	149.9	151.3	150.8	0.453	0.651
Diastolic BP (mmHg)	91.9	92.0	91.9	0.055	0.956
On hypertension medication (%)	57.0%	46.6%	49.8%	1.727	0.085

*Note*. Includes only respondents with both baseline and endline observations

**Table 4:** Changes in Healthy Goals, Behaviors, and Clinical Outcomes from Baseline to Endline by Study Group

	Comparison Baseline	Comparison Endline	Chi-Square (n=94)	Intervention-Baseline	Intervention-Endline	Chi-Square (n=198)
**Healthy Goals**						
Believes is in good health	45.3%	46.3%	0.201 (0.654)	33.8%	72.2%	6.51 (0.011)
Wants to weigh less	52.6%	42.1%	29.8 (0.001)	31.5%	58.9%	24.8 (<0.001)
Believes eats healthy now	21.3%	51.1%	0.012 (0.915)	32.1%	46.9%	1.10 (0.293)
Wants to eat more healthy	45.2%	54.8%	0.164 (0.685)	52.1%	44.3%	0.25 (0.615)
Thinks is sufficiently active	12.9%	50.5%	0.002 (0.968)	33.2%	45.9%	3.51 (0.061)
Wants to be more active	56.5%	51.1%	0.034 (0.855)	61.9%	51.0%	4.00 (0.046)
**Healthy Behaviors last week**			t-test			t-test
Exercise days	3.74	3.92	0.474 (0.636)	3.49	5.55	8.03 (<0.001)
Eating out days	1.27	0.70	2.02 (0.046)	1.49	1.00	3.86 (<0.001)
**Clinical outcomes**						
Hemoglobin A1c (%)	6.49	6.76	0.542 (0.590)	6.52	5.35	3.35 (0.001)
Body Mass Index (Kg/m^2^)	29.1	29.3	0.281 (0.779)	29.2	29.2	0.142 (0.887)
Waist Circumference (cm)	95.8	97.1	0.793 (0.430)	95.0	94.5	0.637 (0.525)
Systolic pressure (mmHg)	149.5	142.7	3.401 (0.001)	151.2	143.1	5.426 (0.001)
Diastolic pressure (mmHg)	92.5	92.8	0.367 (0.375)	92.2	89.9	2.22 (0.100)

**Table 5:** Regression Predictors of Changes in Behavioral and Clinical Outcomes

*Behavior change*	*Model 1: Change in Exercise Days*				*Model 2: Desire for a healthier diet*			
*Predictors (OLS)*	Coef.	Std. Err.	t-test	P>t	Coef.	Std. Err.	t-test	P>t
**Intervention**	**1.826**	0.465	3.92	<.001	**0.644**	0.07	8.64	<.001
Female	0.338	0.524	0.64	0.52	0.018	0.08	0.22	0.83
Age at baseline	0.020	0.019	1.05	0.29	0.004	0.00	1.36	0.18
Overweight or obese	0.166	0.521	0.32	0.75	−0.016	0.08	−0.20	0.84
Diabetes at baseline	0.498	0.450	1.11	0.27	−0.011	0.07	−0.16	0.87
Constant	−1.543	1.330	−1.16	0.25	0.322	0.21	1.57	0.12
			N	257			N	345
			F(5, 251)	3.66			F(5, 251)	15.51
			R-squared	0.068			R-square	0.186
			Adj R-squared	0.049			Adj R-squared	0.174
*Clinical outcome*	*Model 3: Hb A1c below threshold*				*Model 4: Hypertension below threshold*			
*Predictors (Logistic)*	Odds Ratio	95% Conf.	Interval	P>z	Odds Ratio	95% Conf.	Interval	P>z
**Intervention**	**2.307**	1.144	4.653	0.02	**2.672**	0.972	7.345	0.06
Age	0.992	0.964	1.020	0.58	1.011	0.970	1.054	0.59
Female	**0.476**	0.219	1.036	0.06	0.883	0.332	2.347	0.80
Previous diagnosis*	1.375	0.707	2.672	0.35	0.641	0.233	1.760	0.39
Overweight or obese	1.380	0.609	3.129	0.44	**2.852**	0.881	9.234	0.08
More exercise days	0.951	0.861	1.051	0.33	1.009	0.906	1.124	0.87
Constant	0.919	0.135	6.276	0.93	0.105	0.006	1.804	0.12
								
			N	162			N	112
			LR chi2(6)	10.12			LR chi2(6)	7.43
			Pseudo R2	0.045			PseudoR2	0.050

* Diabetes diagnosed for Diabetes model, Hypertension diagnosed for Hypertension model

## Data Availability

Data that support the findings of this study are available in Figshare with the identifier https://doi.org/10.6084/m9.figshare.28388258.

## Abbreviations

BMI: Body Mass Index
BP: Blood pressure
CDC: Centers for Disease Control and Prevention
CHW: Community health worker
CSCOM: “Centre de Santé Communautaire” (Community health centers)
CVD: Cardiovascular diseases
DBP: Diastolic blood pressure
DPP: Diabetes Prevention Program
DPP-P2P: Diabetes Prevention Program’s Power to Prevent
EDTA: Ethyldiaminetetraacetic Acid
HbA1c: Hemoglobin A1c
IDEA: Iterative Decision-making for Evaluation of Adaptations
OLS: Ordinary Least Squares
OMR: Optical Mark Recognition
PE: Peer educator
PPD-Mali: ‘Programme de Prévention du Diabète au Mali’ (Diabetes Prevention Program in Mali)
SSA: Sub-Saharan Africa
SSBR: Small Steps, Big Rewards
SST: Serum separator tube
SBP: Systolic blood pressure
US: United States
