# Supplementary material for: A Hybrid Effectiveness-Implementation Trial of the ‘Power To Prevent Diabetes Program’ in Bamako, Mali
Source: Glob Implement Res Appl. Author manuscript; Available in PMC 2026 Feb 5. (PMC12872172; doi:10.1007/s43477-025-00199-x)
Supplement: Exercise booklet [file NIHMS2139094-supplement-Exercise_booklet.pdf]

Université des Sciences, des Techniques et des Technologies de Bamako

FACULTE DE MEDECINE ET D'ODONTO-STOMATOLOGIE
FMOS
BAMAKO - MALI

DEPARTEMENT D'ENSEIGNEMENT ET RECHERCHE EN SANTÉ PUBLIQUE
DERSP
BAMAKO
MALI

DES ACTIVITÉS PHYSIQUES QUE VOUS POUVEZ INCORPORER DANS VOTRE VIE

Les guides du Programme de Prévention du Diabète et d'Hypertension au Mali

(PPD- Mali)

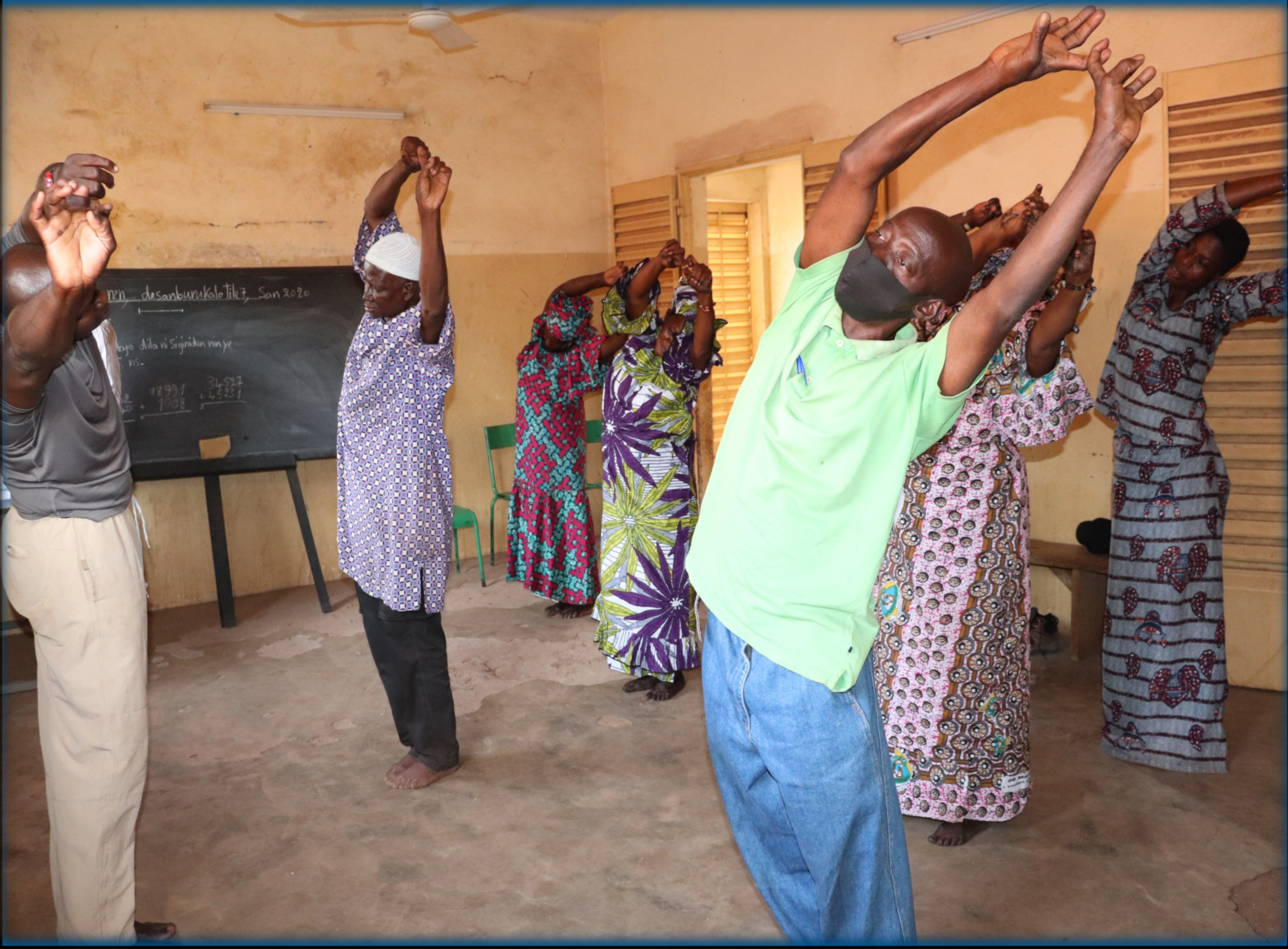

Sommaire

I. INTRODUCTION……………………………………………………………3

II. COMPOSANTS DU LIVRET……………………………………………3

A. *DESCRIPTION DES EXERCICES*…………………………………4,5,6,7,8

1. ETIREMENT…………………………………………………………4,5

a. Etirement vertical……………………………………………………4

b. Etirement Latéral……………………………………………………4

c. Mouvements des Reins……………………………………………4

d. Mouvement de la Hanche…………………………………………5

2. RENFORCEMENT…………………………………………………5,6,7

a. Marche de Face ou Marche de Statique…………………………5

b. Renforcement Abdominal Simple…………………………………6

c. Renforcement Abdominal Modéré…………………………………6

d. Renforcement Abdominal Vigoureux………………………………6

e. Travail du Bassin……………………………………………………7

f. Travail des Bras……………………………………………………7

g. Rotation des Bras……………………………………………………7

3. RECUPERATION……………………………………………………8

a. Etirement des Pieds…………………………………………………8

b. Rotation des Bras……………………………………………………8

c. Rotation de la Hanche………………………………………………8

B. *MENU DES EXERCICES*……………………………………………9

PROGRAMME DE PRÉVENTION DU DIABÈTE ET D'HYPER-TENSION AU MALI

(PPD- MALI)

I. Introduction

Programme de Prévention du Diabète et d' Hypertension: Les activités physiques pour une meilleure santé

Au cours des dernières décennies, les taux de diabète et de maladies cardiovasculaires ont augmenté rapidement en Afrique subsaharienne. Au Mali en 2010, une glycémie élevée ou un diabète diagnostiqué a été trouvé chez 5,6% des femmes et 6,3% des hommes, tandis que 26% étaient hypertendus.

L'accès aux soins et aux médicaments pour le diabète et l'hypertension est limité et coûteux, et beaucoup de cas restent non diagnostiqués ou non contrôlés. Les déficits dans la disponibilité des traitements soulignent l'importance de la mise en œuvre de la prévention communautaire et des stratégies de prise en charge des maladies cardiovasculaires, mais le traitement des maladies cardiovasculaires à ce jour n'est basé que sur le traitement médicamenteux, aucun programme communautaire ne soutenant des changements complémentaires au régime alimentaire et à l'exercice physique.

C'est ainsi que l'Universités des Sciences, des Techniques et des technologies de Bamako (**USTTB**), en collaboration avec l'Université de Columbia et **HPII** aux **USA** mettent en place un programme de prévention de diabète (PPD) dans les quartiers périphériques de Bamako.

Ce programme est une adaptation du « Diabètes **Prévention Program (DPP**) » des Etats Unis d'Amériques, qui s'est avéré plus efficace que les médicaments seuls ou les programmes de changement de style de vie en clinique.

II. Composants du Livret

Ce livret:

Contient des exercices physiques recommandés, dans le cadre de la prévention de diabète et l'hypertension, bénéfiques en dépenses calorifiques. Ils peuvent s'effectuer seul, avec un partenaire ou en groupe, à domicile (***chambre, salon, terrasse, cours,)*** ou dehors ***(rue, espace public, terrain de sport….).***

A. Description des Exercices

Il s'agit de trois catégories d'activités physiques:

1. <u>Etirement</u>: Le but c'est de préparer les muscles et le corps pour des activités plus intenses, généralement pour une durée de 5-10 minutes.

a. <u>Etirement Vertical</u>**:** Se mettre sur les **bouts de pieds** les mains en l'air chercher le plus haut possible. Compter 10X3;

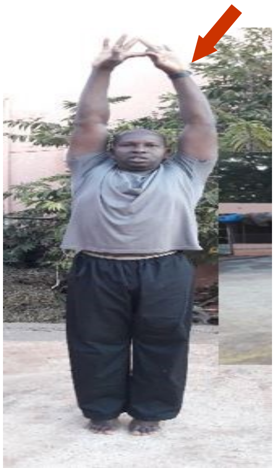
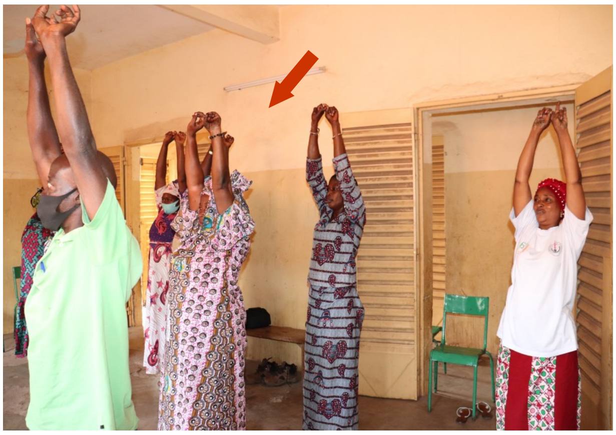
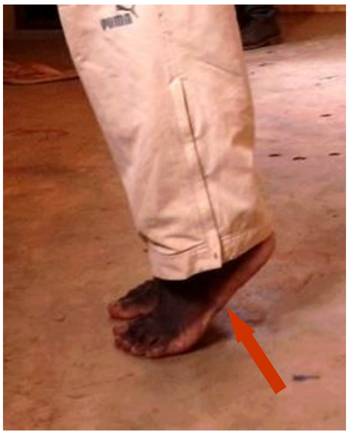
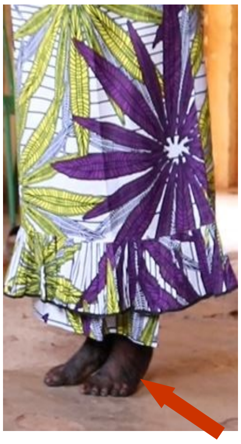

b. <u>Etirement Latéral</u>**:** Ecarter légèrement les jambes, compter 10, cassé à gauche, compter 10, cassé à droite.10X3;

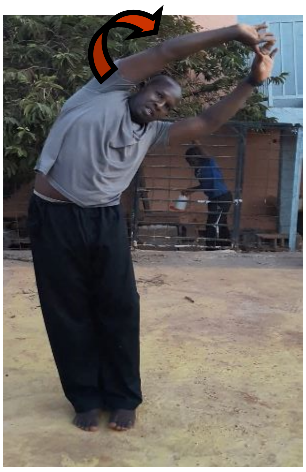
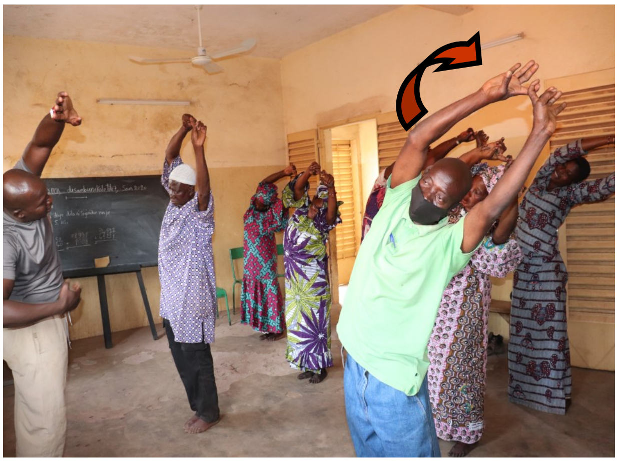
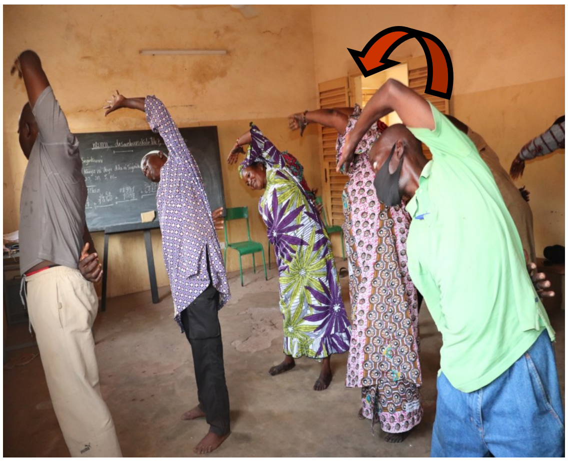

c. <u>Mouvement des reins</u>: **Les mains au bassin**, faire 10 rotations à droit et à gauche(.10X3);

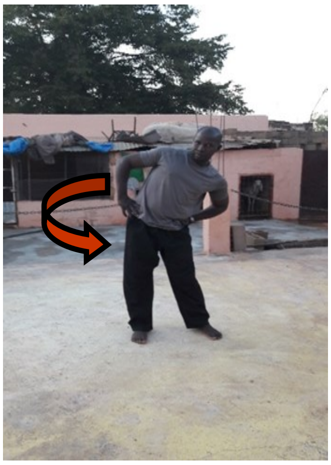
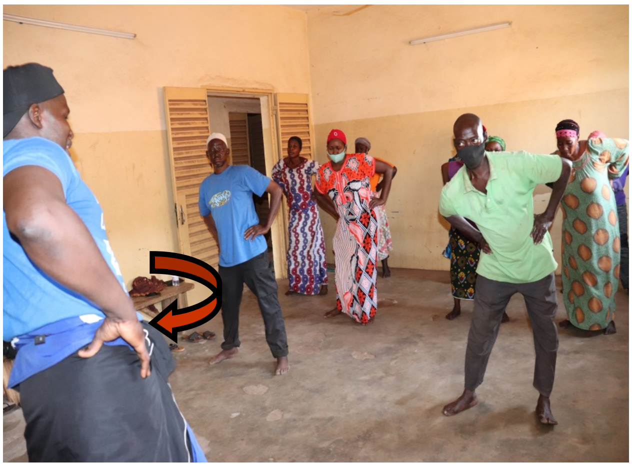
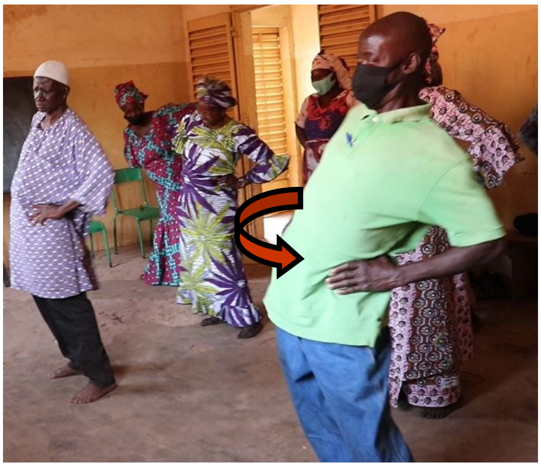

d. Mouvement de la Hanche : Se mettre en position debout, les pieds écartés à la largeur des épaules, **bras droit au dessus de la tête, bras gauche au dot, cassé le bassin à gauche**, compter 10, répéter la même chose dans le sens contraire.

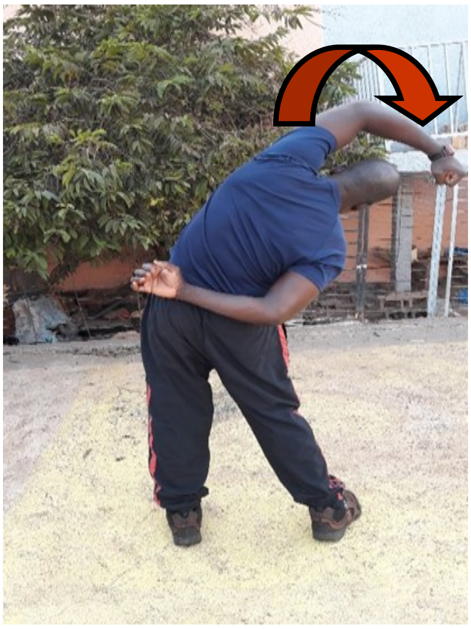

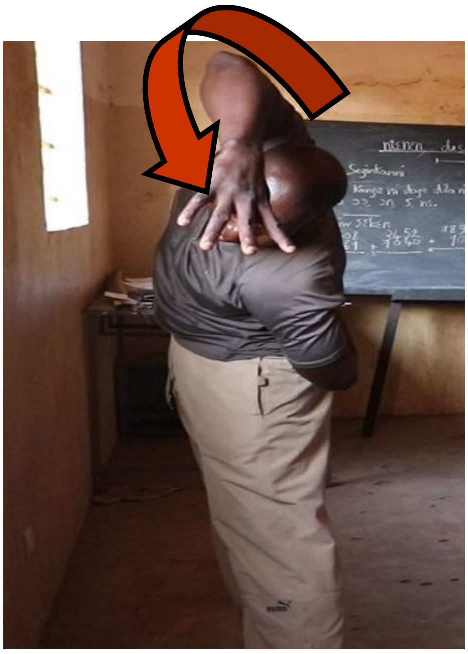

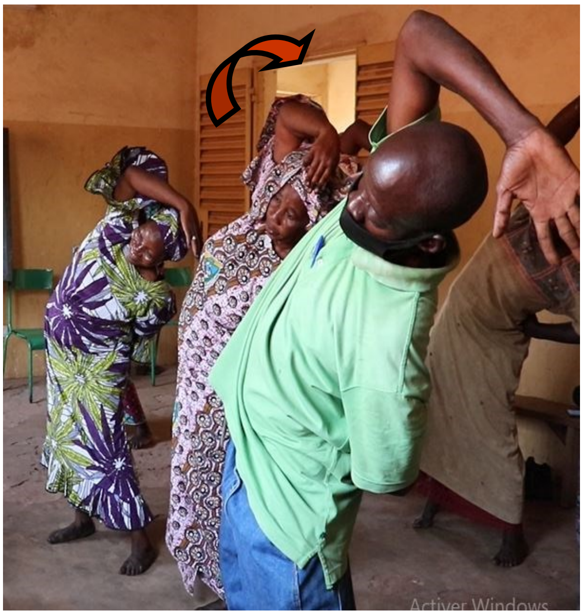

2. Renforcement: Ces activités sont destinées au renforcement des muscles de votre corps, souvent avec plusieurs exercices, chacun pour un aspect du corps; généralement, pour 15-30 minutes.

a. Marche Statique: Sur place et Suivant les quatre points cardinaux, Compter **100 PAS de chaque coté**, alterné de pause de 15 inspirations et expirations profondes. Elle peut aussi s'effectuer en déplacement (sur 1km).

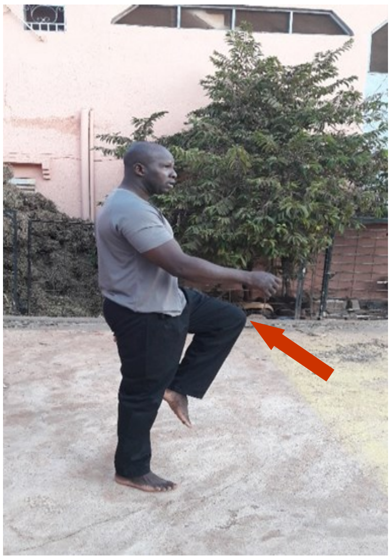

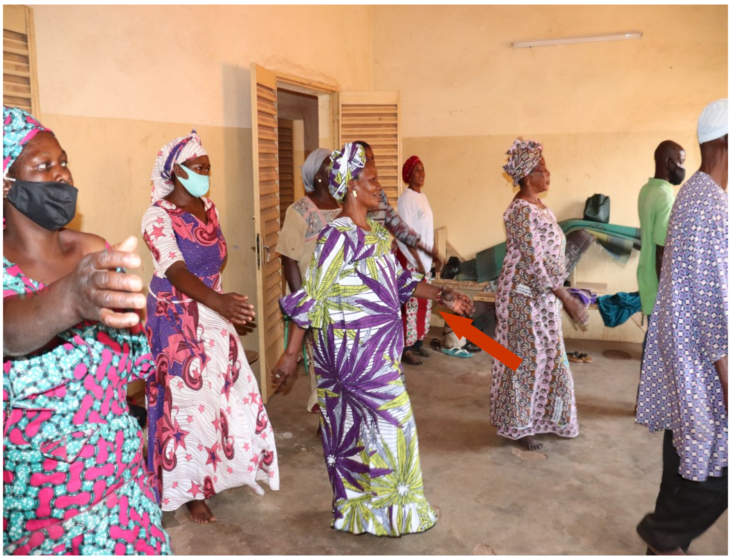

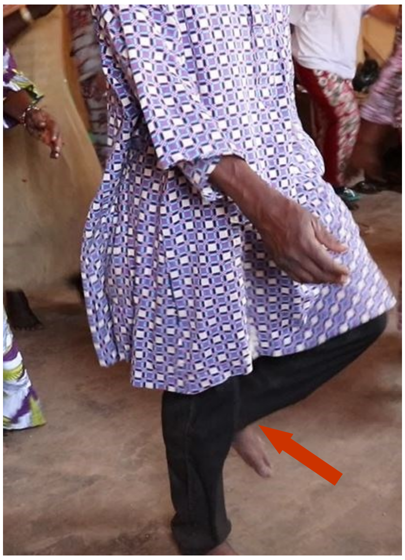

b. <u>Renforcement Abdominal Simple</u>**: se mettre sur le dos**, sur les deux coudes. **Soulever un pied**, compter 10, changer de pied, compter 10. Mouvement à répéter trois fois moins;

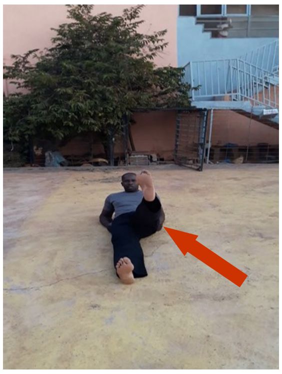
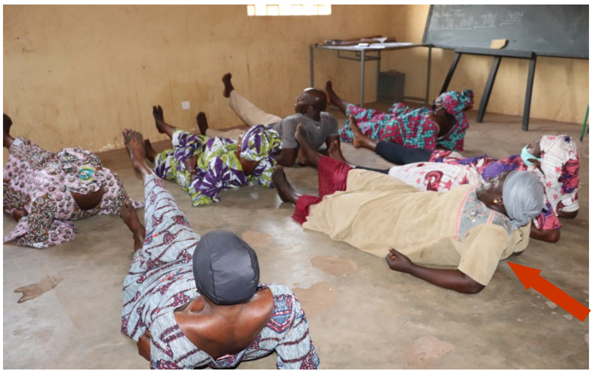
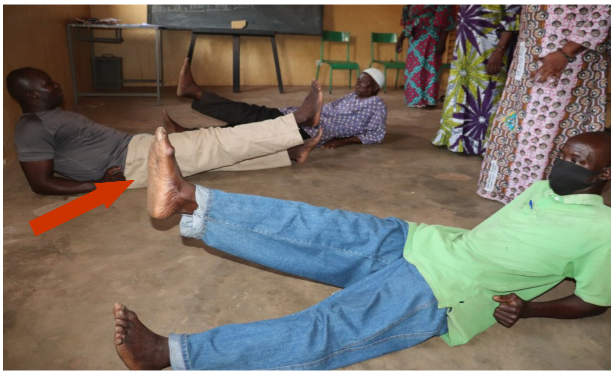

c. <u>Renforcement Abdominal Modéré</u>: **Se mettre sur le dos,** appui sur les coudes, **deux pieds joints, repliés tendus**, répéter 10 fois 3.

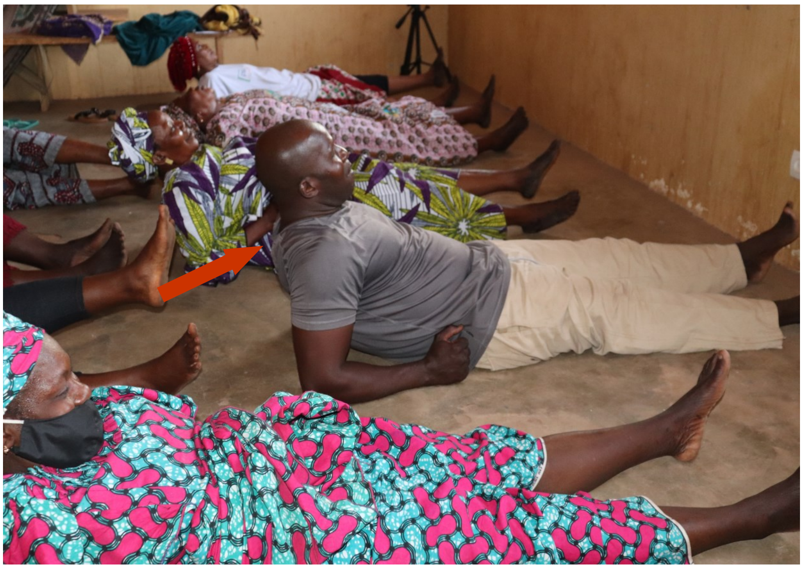
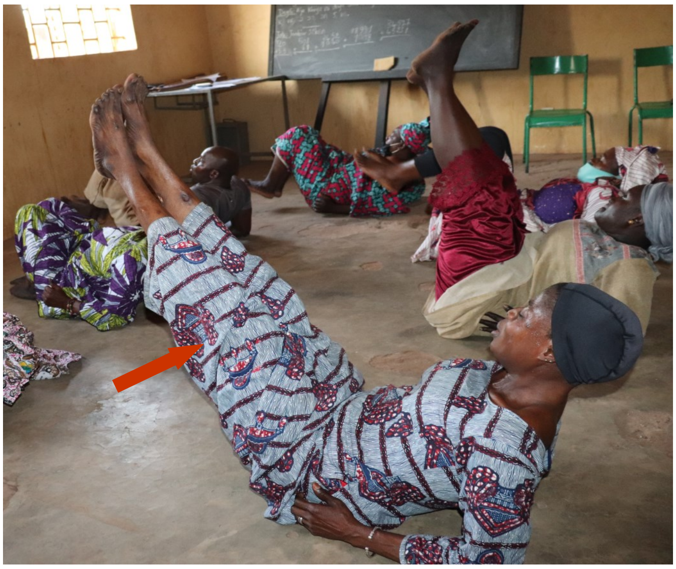

d. <u>Renforcement Abdominal Vigoureux</u>: Etre en duo, **l'un couché sur le dos, les pieds repliés bloqués par et l'autre en position debout, les bras tendus, paumes face au partenaire au sol**; l'objectif: **taper dans les paumes du partenaire debout et revenir en position standard**. Répéter 10 fois. Puis changer le rôle entre partenaires, trois fois.

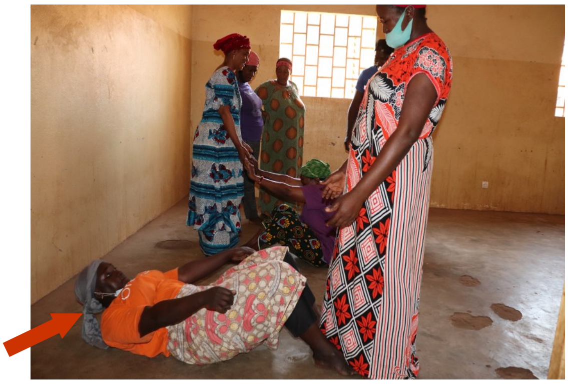
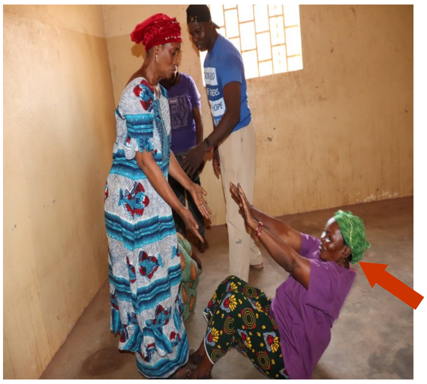
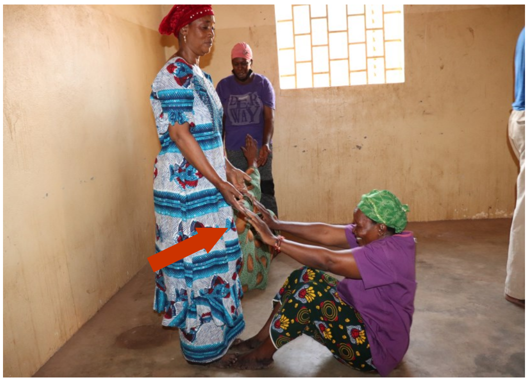

e. Travail du Bassin: se mettre à quatre pattes, soulever un genou à la hauteur du bassin, puis ramener, compter 10 coté droit et 10 coté gauche.

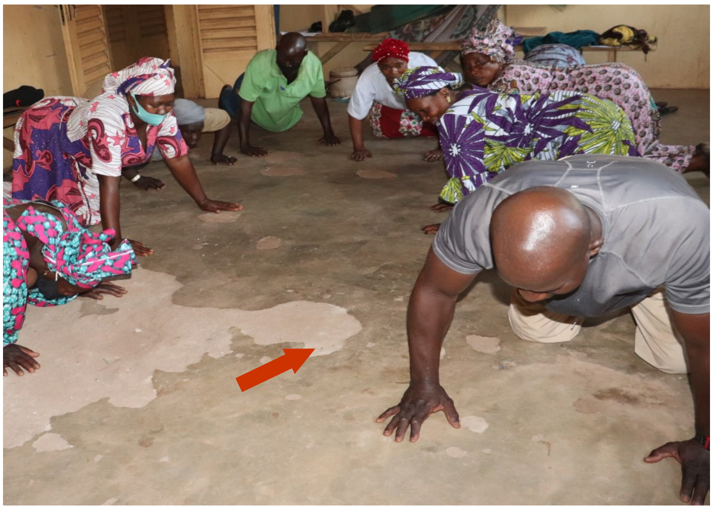
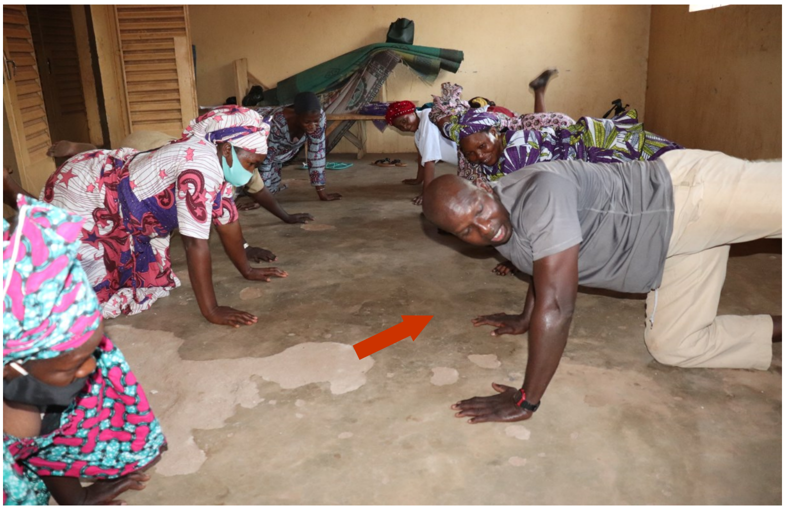

f. Travail des Bras: **Se mettre à quatre pattes, fléchir sur les bras**, la tête le plus loin possible, compter 10 fois 3, alternées de pause. Même exercice en **décollant un genou du sol**, 10 fois pour chaque genou.

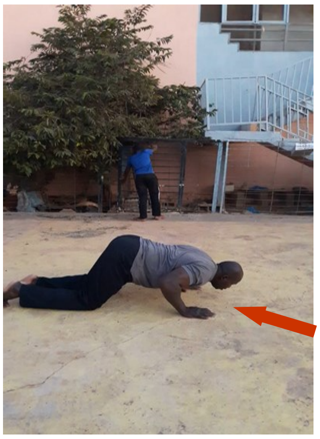
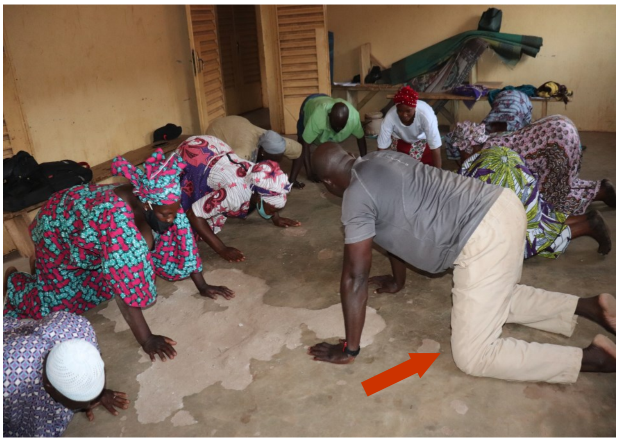
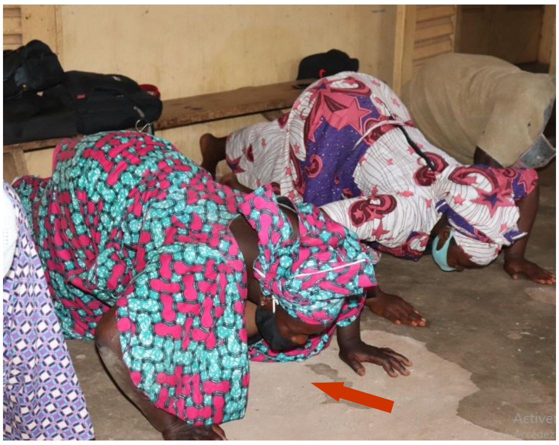

g. Rotation des Bras: Faire roter **les deux bras repliés en avant** 10 fois, et 10 le contraire.

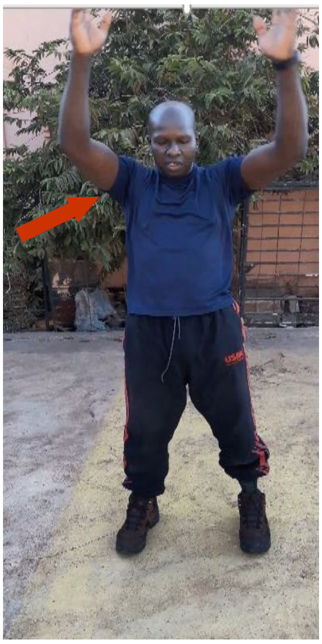
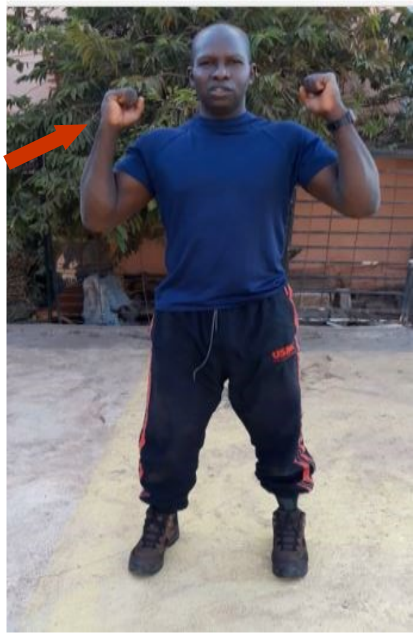
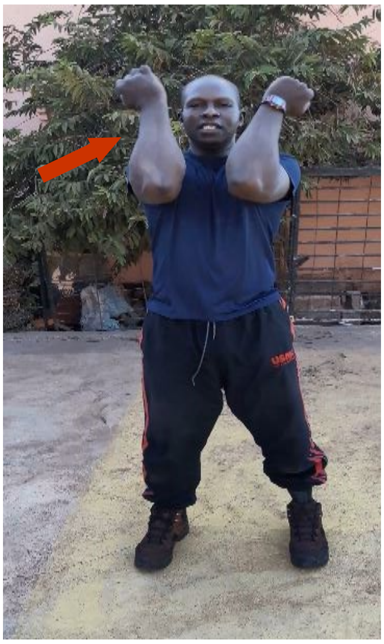
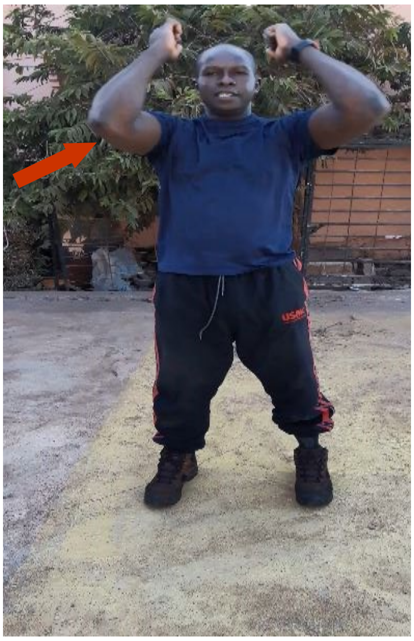

3. <u>**Récupération:**</u> **Le but est de mettre le corps à l'aise après les exercices de renforcement, généralement pour 5 minutes.**

a. <u>Etirement des pieds</u>: Se mettre au PAS de marche, **soulever le bout du pied** avant et l'attraper avec les deux mains. Ensuite changer de pieds et répéter la même chose. 2 à 3 fois.

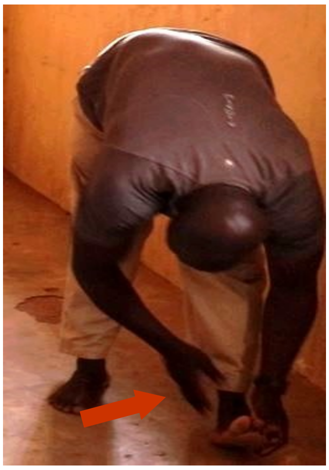
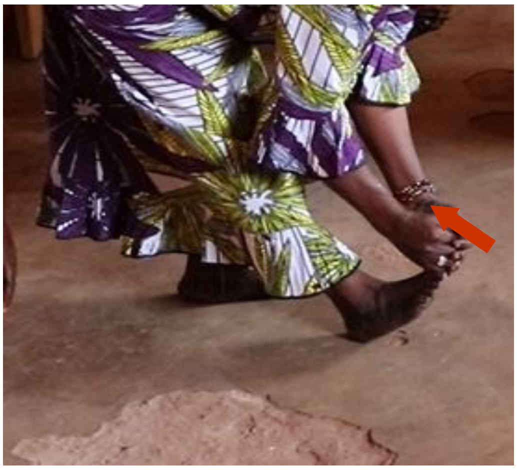
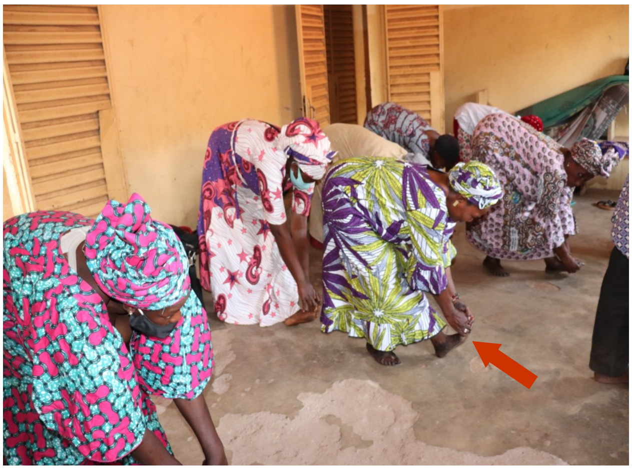

b. <u>Rotation des genoux</u>: Faire roter **les genoux** dans un sens et dans l'autre.

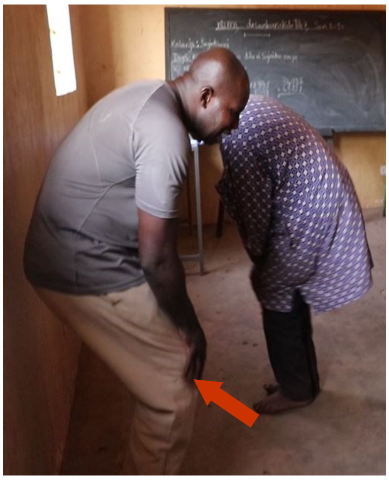
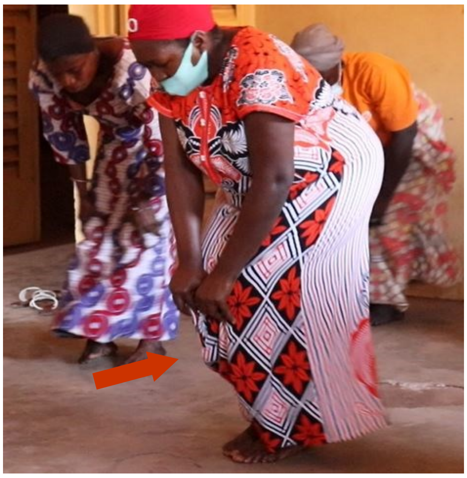
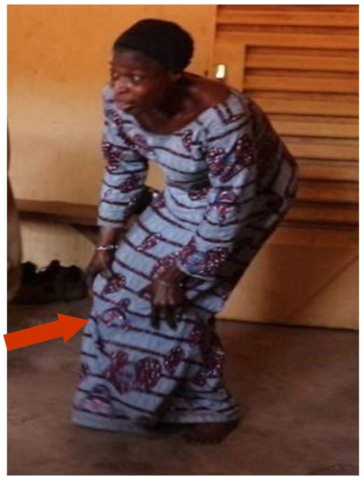

c. <u>Rotation de la hanche</u>: Placer les mains sur la hanche, puis faire roter **la hanche** dans un sens et dans l'autre.

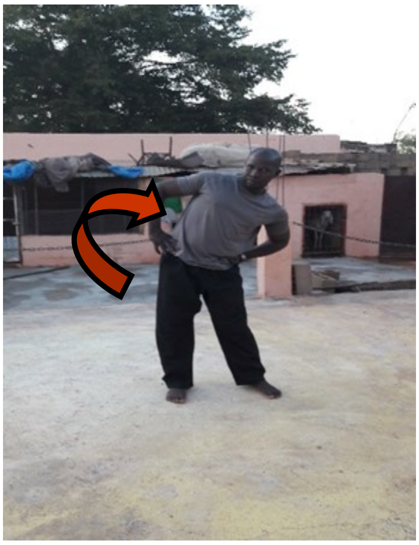
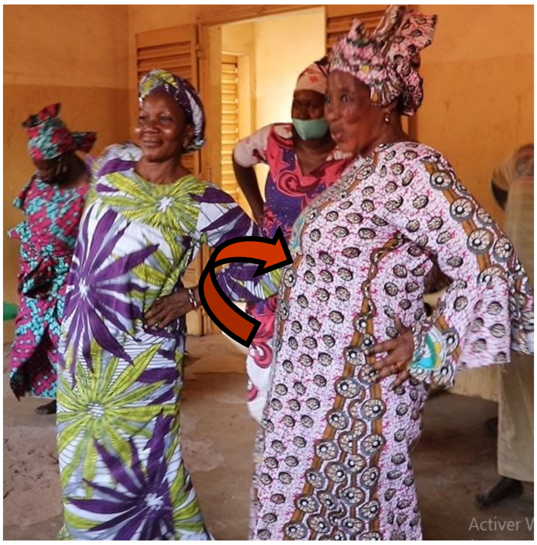
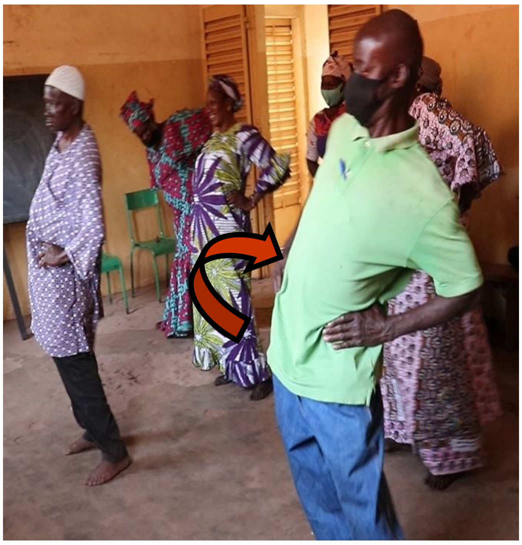

B. Menu des Exercices

CATEGORIES D'EXERCICES	SIMPLE (Sans essoufflement)	MODERE (Un peu essoufflé, on peut parler clairement)	VIGOUREUX (Essoufflé, difficultés de parler pendant l'exercice)
ETIREMENT (3-5*min)*	1.a. ou A.1.b.	1.b. ou A.1.c.	1.c. ou 1.d.
RENFORCEMENT (20-25*min)*	2.a. ou 2.b.	2.b. ou 2.e. ou 2.f.	2.c. ou 2.d.
RECUPERATION (3-5*min)*	3.a.ou 3.b.	3.a. ou 3.b.	3.b.ou 3.C.
PAS	**5000**	**7500**	**10 000**
DUREE PAR JOUR	***20-30 minutes***	***45 minutes***	***60 minutes***
JOURNEES PAR SEMAINE	***3X***	***3x simple*** ***2X modéré***	***3x vigoureux*** ***2X modéré***
