# Supplementary material for: A Hybrid Effectiveness-Implementation Trial of the ‘Power To Prevent Diabetes Program’ in Bamako, Mali
Source: Glob Implement Res Appl. Author manuscript; Available in PMC 2026 Feb 5. (PMC12872172; doi:10.1007/s43477-025-00199-x)

MODE DE PRÉPARATION DES DIFFERENTES SAUCES MALIENNES ADAPTÉES AU PPD

Guide du Programme de Prévention du Diabète et d'Hypertension au Mali (PPD- Mali)

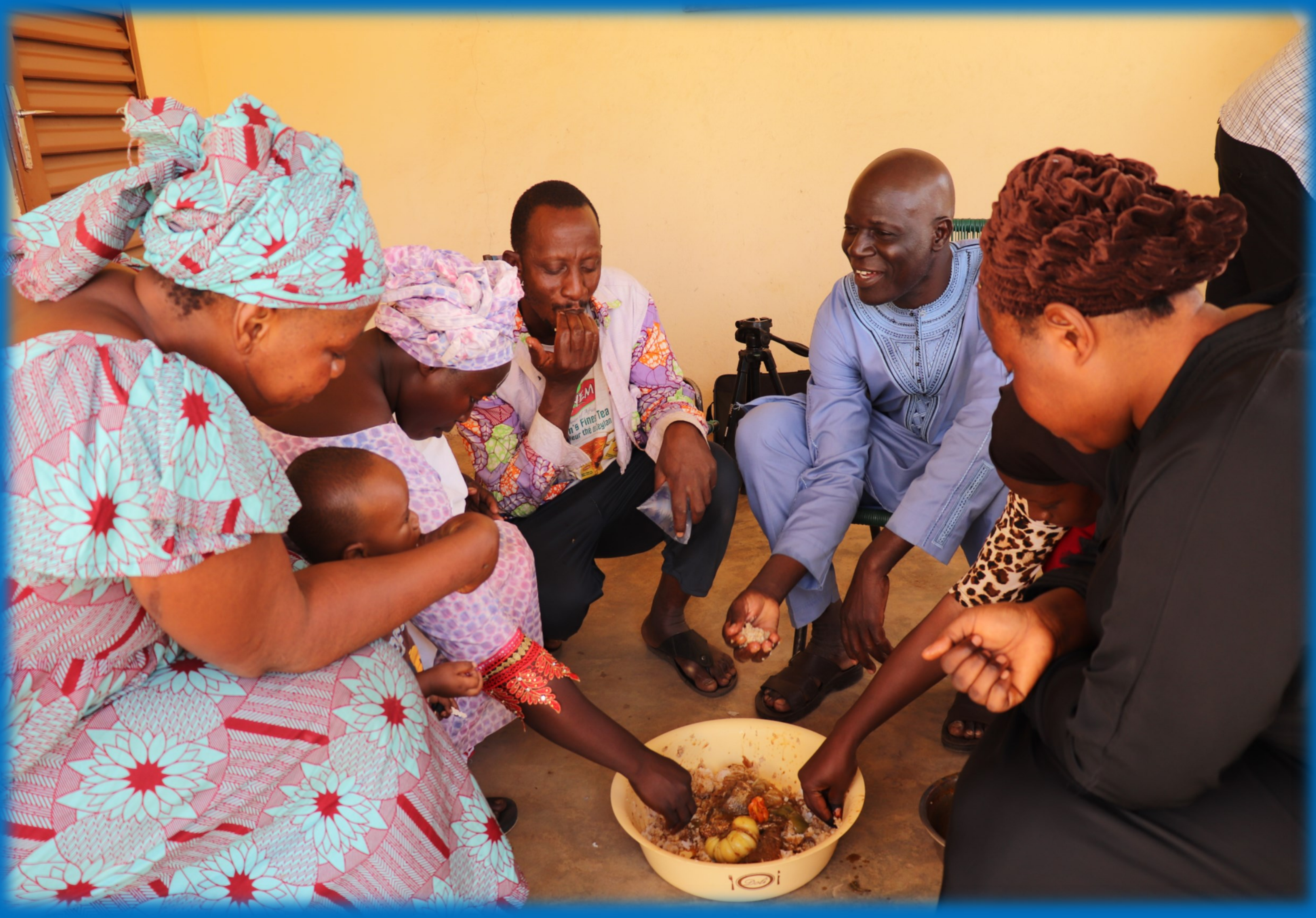

Sommaire

I. INTRODUCTION..2

A. ***DESCRIPTION : MODES DE PREPARATION DES SAUCE***........................3

* RECETTE **I:** SAUCE DIABADJI...3,4
* RECETTE **II :** SAUCE TIGUADEGUE 5,6
* RECETTE **III:** SAUCE SAKA SAKA 7
* RECETTE **IV:** SAUCE GOMBO FRAIS...8
* RECETTE **V :** SAUCE FAKOYE...10
* RECETTE **VI:** ZAME...11
* RECETTE **VII:** SAUCE YASSA 12

PROGRAMME DE PRÉVENTION DU DIABÈTE ET D'HYPERTENSION AU MALI

(PPD- MALI)

I. Introduction

Programme de Prévention du Diabète et d' Hypertension:

MODE DE PRÉPARATION DES DIFFERENTES SAUCES MALIENNES ADAPTÉES DU PPD

Au cours des dernières décennies, les taux de diabète et de maladies cardiovasculaires ont augmenté rapidement en Afrique subsaharienne. L'accès aux soins et aux médicaments pour le diabète et l'hypertension est limité et coûteux, et beaucoup de cas restent non diagnostiqués ou non contrôlés. Les déficits dans la disponibilité des traitements soulignent l'importance de la mise en œuvre de la prévention communautaire et des stratégies de prise en charge des maladies cardiovasculaires.

C'est ainsi que l'Universités des Sciences, des Techniques et des technologies de Bamako (**USTTB**), en collaboration avec Columbia University et Health Partners International Institute (**HPII**) aux USA mettent en place un programme de prévention de diabète (PPD) dans les quartiers périphériques de Bamako.

Ce programme est une adaptation du « Diabètes **Prévention Program (DPP**) » des Etats Unis d'Amériques, qui s'est avéré plus efficace que les médicaments seuls ou les programmes de changement de style de vie en clinique.

Ce livret contient des types de sauces maliennes adaptées aux recommandations dans le cadres du programme de prévention du diabète et de l'hypertension bénéfiques à l'équilibre calorifique et de la glycémie. Pour chaque sauce, les méthodes de préparation sont adaptées pour réduire la quantité de la graisse et du sel.

- **Enlever d'abord la graisse de la viande avant de la cuire;**
- **Mesurer l'huile avec une cuillère pour limiter la quantité d'huile dans la sauce;**
- **Réduire la quantité du sel**
- **Ajouter plus de légumes dans toutes les sauces!**

A. Description des Modes préparations des sauces

Il s’agit de cinq types de sauces étudiées et adaptées:

1. RECETTE : « SAUCE DIABADJI », Avec viande de bœuf, pour 6 personnes

a. INGRÉDIENTS :

½ kg de viande ou une quantité à convenance;

3-4 petites cuillérées d’huile d’arachide;

8 oignons de grande taille;

3-4 tomates fraiches ;

Mélange des légumes : choux, aubergines, piments, courgettes, poivron ou d’autres;

2 grandes cuillérées de concentrée de tomate;

1 petite cuillérée de sel d’un sachet de 25F ;

Les épices I : 1 petite cuillérée de poudre de soumbala, 2 grandes cuillerées d’oignons frits en poudre, 1 gros morceau de poisson séché;

Les épices II : 1 gousse d’ail de taille moyenne, hachées; 2 grandes cuillérées de feuilles de persil, d’oignon et poivrons pilés ; poivre;

Les épices III : ½ petite cuillérée poudre de gombo.

b. ETAPE 1 : LES PRÉPARATIFS :

- **Enlever toutes les graisses visibles en lavant la viande, apprêter les légumes et les 3 groupes épices.**

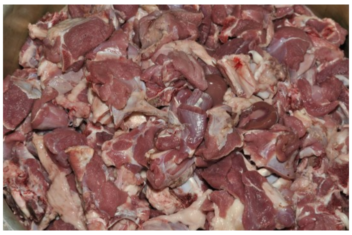
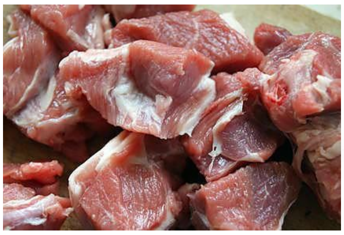
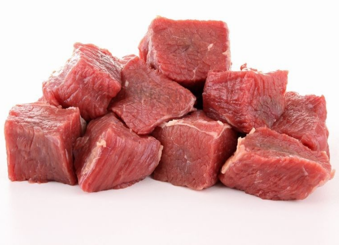
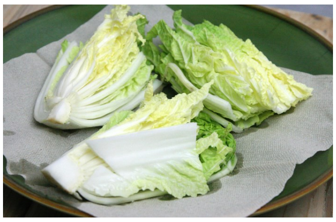
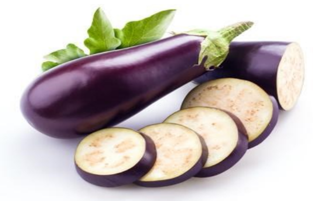
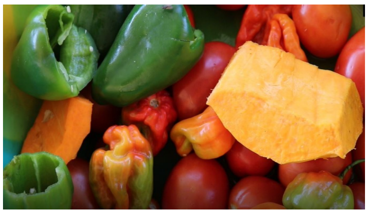
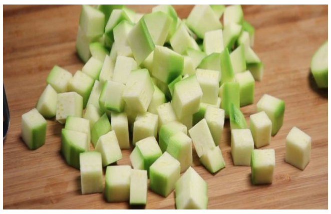
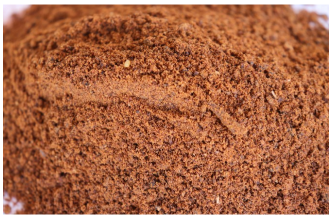
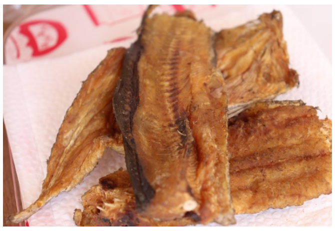
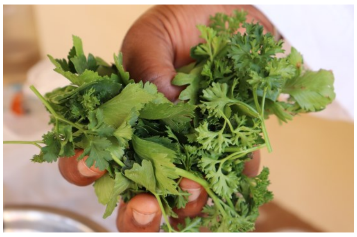
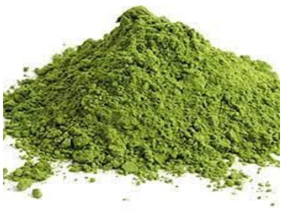
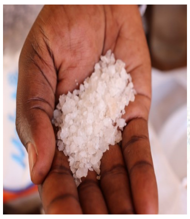
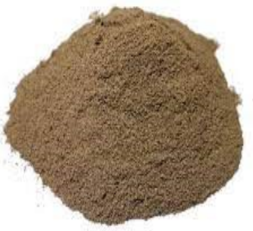

c. ETAPE 2 : LA CUISSON :

- Chauffer l'huile puis mètre la viande et les oignons frais, et le premier groupe d'épices et les tomates. La laisser mijoter après l'avoir remué, jusqu'à ce que les oignons fondent complètement.

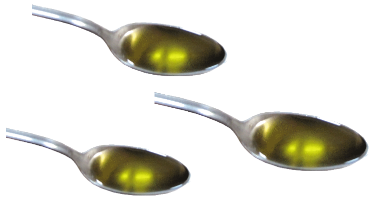
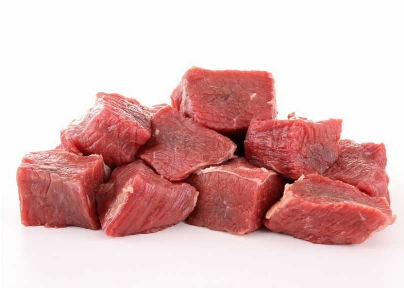
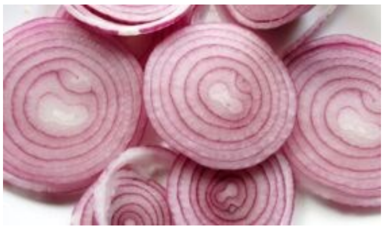
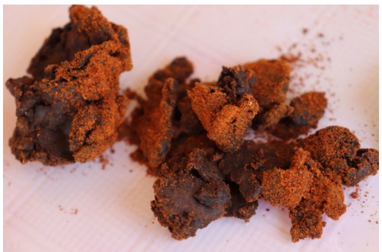
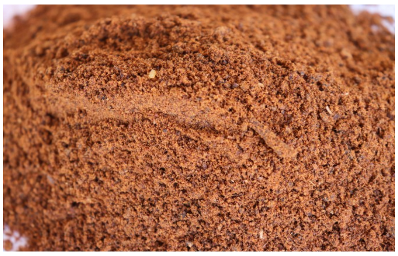
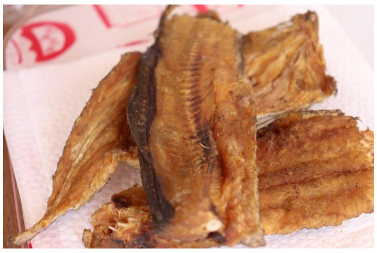
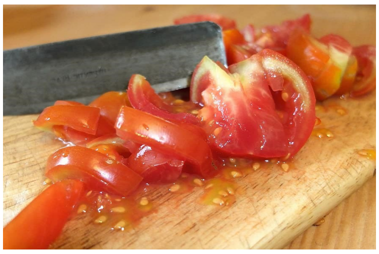
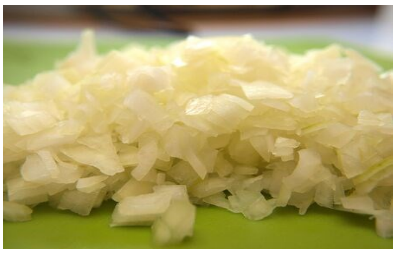
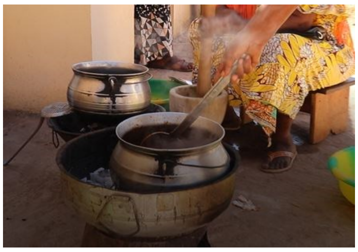

d. ETAPE 3 : LA FINITION :

- Ajouter les autres légumes ainsi que les épices III. Puis, ajouter de l'eau pour une sauce plus épaisse.

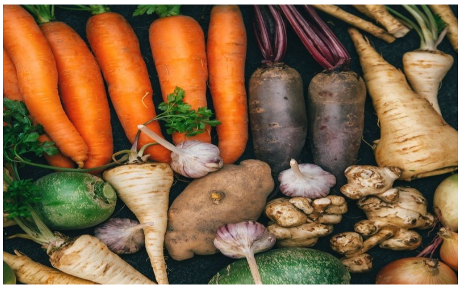
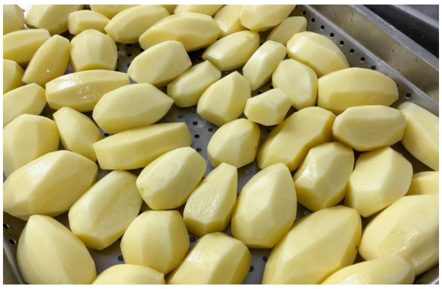
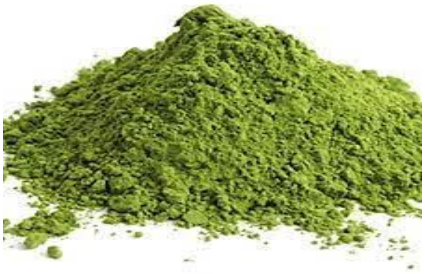
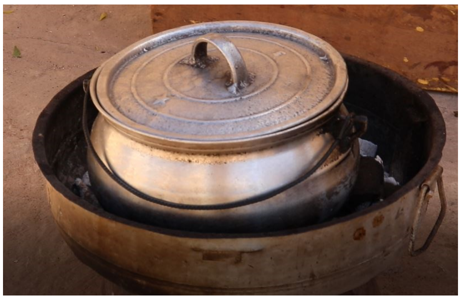
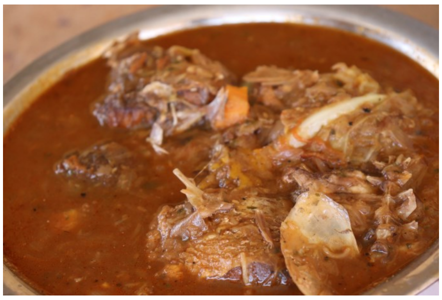
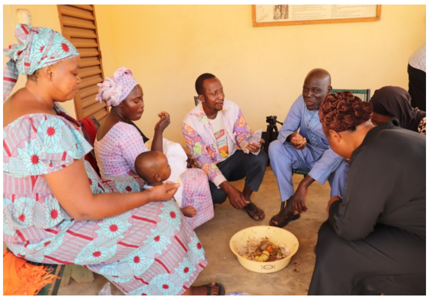

- « SAUCE DIABADJI », **à base de poisson frais ½ kg ou de 250 à 1000 F , pour 6 personnes.**
- Assaisonner les poissons avec du poivre, de l’ail et du persil pilés. Mettre le poisson, 1-2 morceaux à la fois, dans la marmite avec une cuillère d'huile chaque fois. Cuire pour 2 minutes, puis enlever les morceaux et répéter pour tout les poissons. Continuez comme dans la sauce à viande.

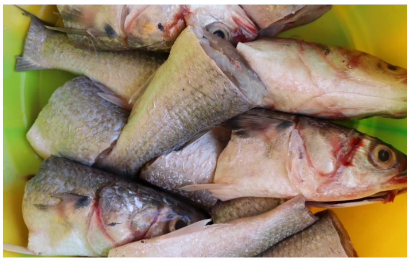
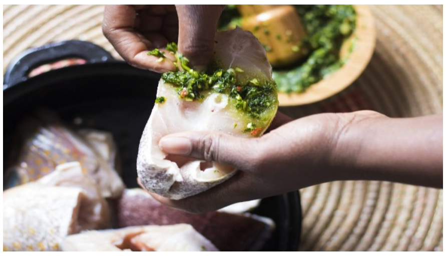
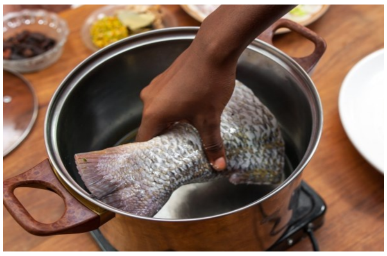
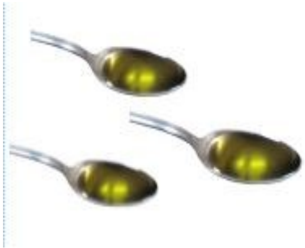
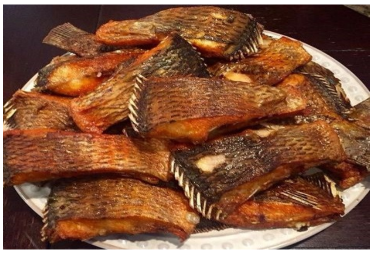
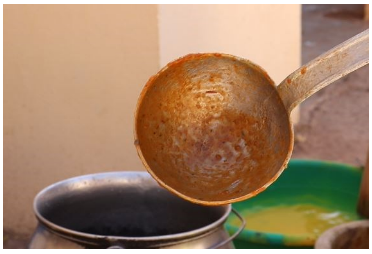
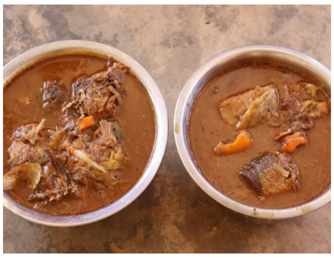

2. **RECETTE:** « SAUCE TIGUADEGUÉ », Avec la viande du bœuf, pour 6 personnes.

a. INGRÉDIENTS :

- ½ kg de viande de bœuf ou une quantité;
- Oignon frais;
- Une louche de pate d'arachide;
- 2 grandes cuillérées de poisson séché en poudre;
- 2 tomates fraîches;
- 1 cuillérée de concentrée de tomate;
- 1 gousse d'ail;
- 1 petite cuillérée de soumbala;
- 1 grande cuillérée oignon frit;
- Légumes : choux, aubergines, piments frais, courgettes, poivron et d'autres légumes;
- 1 petite cuillerée de sel d'un un sachet de 25F
- 3-4 Gombo (estimés entre 50 et 100F) ou à convenance.
- Poivre et lauriers à volonté

b. ETAPE 1 : LES PRÉPARATIFS :

- **Découper les tomates fraîches, les oignons, l'ail, les légumes, et apprêter les épices.**

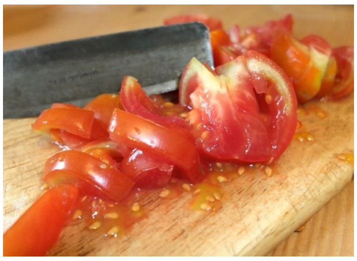
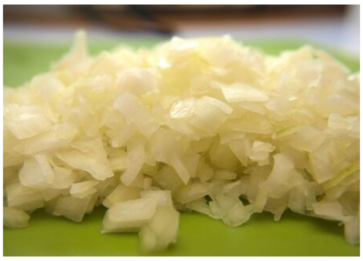
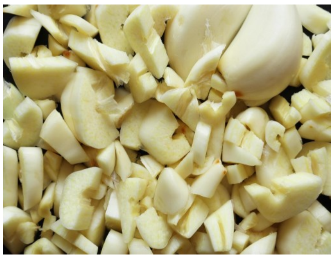
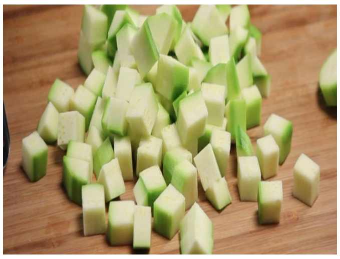
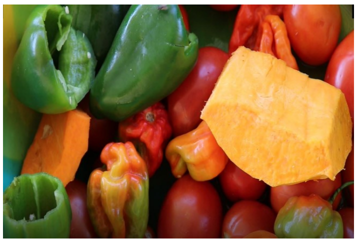
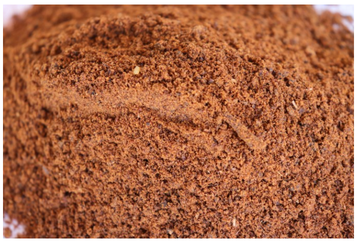
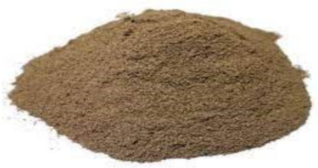
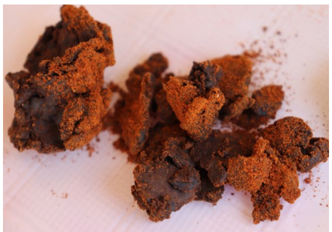

c. ETAPE 2 : LA CUISSON:

- **Mettre de l'eau, puis les oignons frais, l'ail, les tomates fraiches écrasées, pate de tomate dans la marmite. Ensuite, la pâte d'arachide mélangée de la poudre de Soumbala, d'oignon frits et le poisson séché. Après de 10 minutes, assaisonner avec le reste des épices, le mélange de légumes frais.**

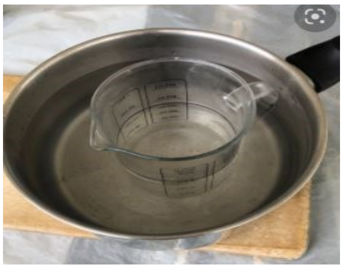
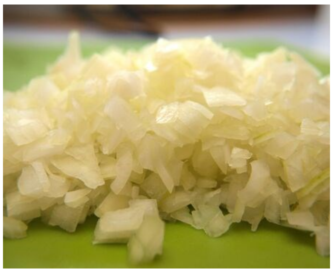
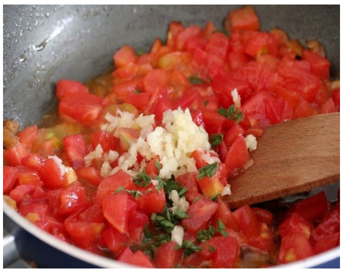
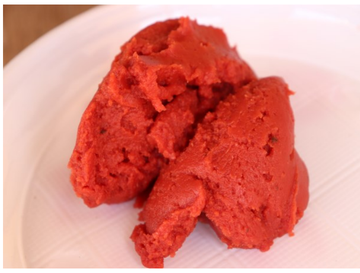
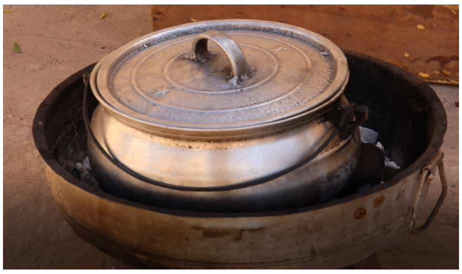
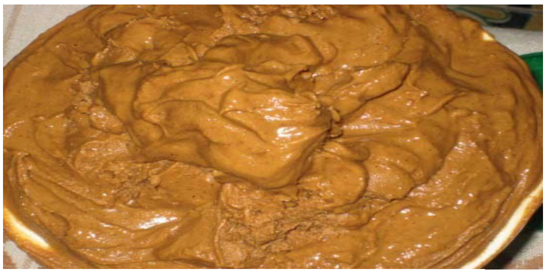
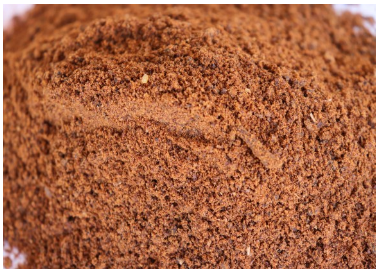
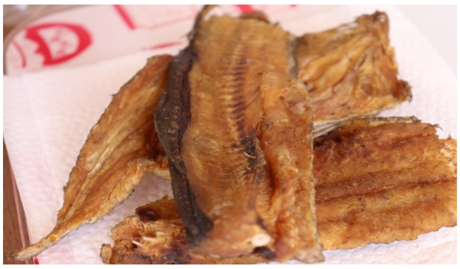
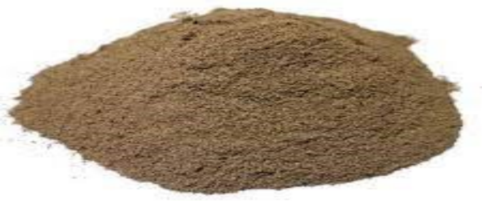
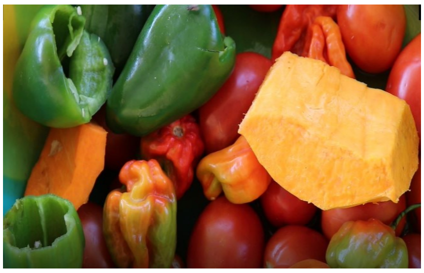

d. ETAPE 3 : LA FINITION :

- **Ajouter le gombo frais, pour agrémenter la sauce.**

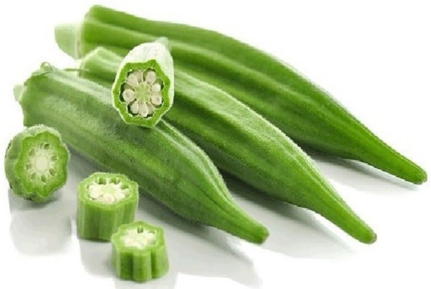
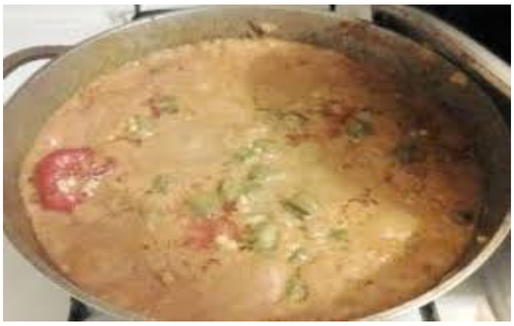
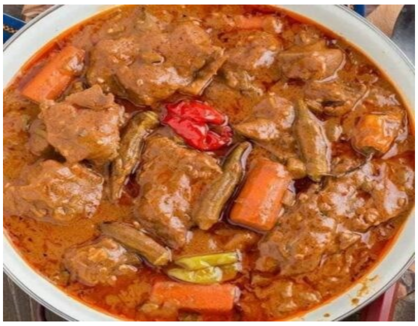

- **«TIGUADEGUE» à base de poisson fumé : le mode de préparation relativement est identique, avec l'addition des poissons fumés à convenance.**

3. <u>RECETTE</u>: «SAUCE SAKA SAKA» à feuille de Patate Douce», Avec la viande de bœuf, pour six personnes.

a. <u>INGRÉDIENTS</u> :

- ½ kg de viande;
- Feuilles de patate douce;
- 4 tomates Tomate fraiches;
- 3 oignon frais;
- Poivron, si nécessaire
- Aubergines locales, feuilles d'oignon et piments;
- 2 petites cuillérées de poudre Soumbala;
- 2 Cuillérées de poudre séché ou wouloudjègué;
- 4 grandes cuillérées d'huile d'arachide;
- 4 morceaux de gombo frais si nécessaire;
- 1gousse d'ail; Poivre;
- 1 petite cuillère de sel d'un sachet de 25F

b. <u>ETAPE 1 : LES PRÉPARATIFS</u> :

- **Enlever la graisse de la viande, couper les légumes et préparer les épices.**

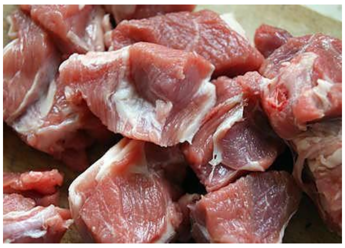
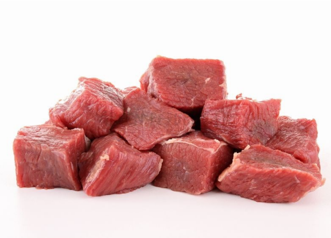
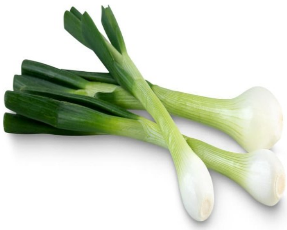
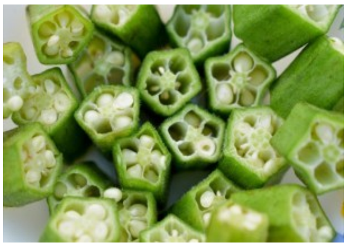
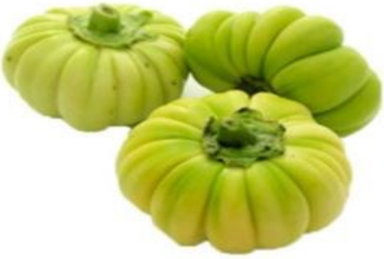
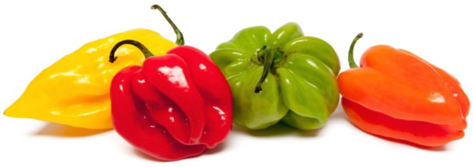
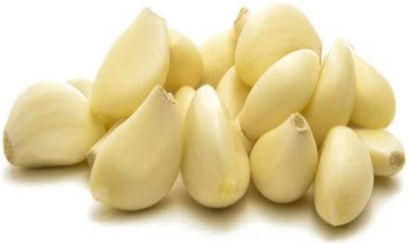
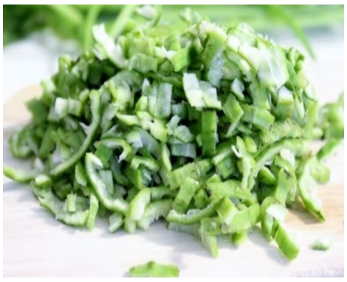
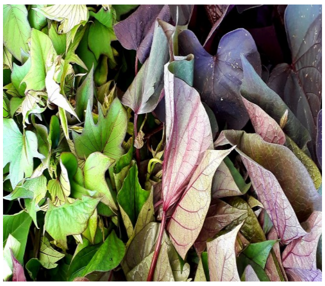
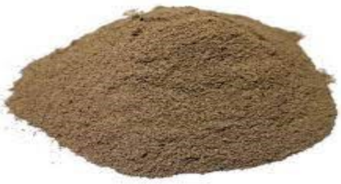
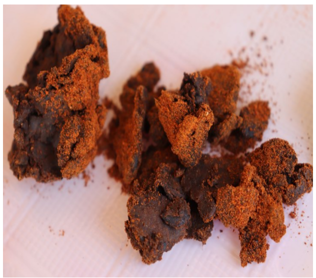

c. ETAPE 2 : LA CUISSON:

- Mettre l'huile d'arachide dans la marmite et ajouter la viande. Remuer et chauffer pour 10mn, puis ajouter les oignons frais, le poivron, les tomates fraiches. Remuer encore 10mn. Ensuite, les feuilles de patate douce et d'oignon, l'ail, les épices, de poisson séché et le sel, les aubergines locales, le gombo et le piment frais. Remuer et ajourer de l'eau.

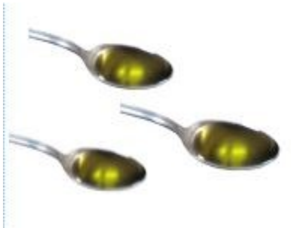
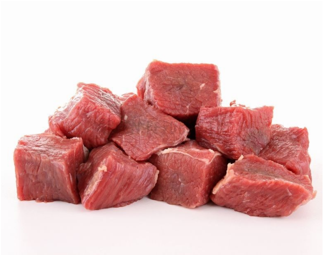
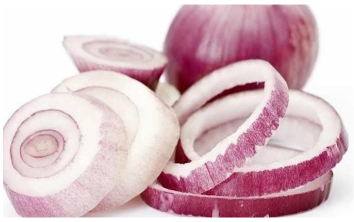
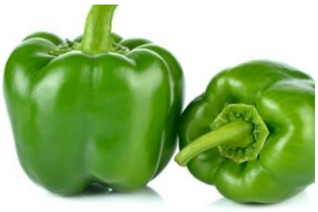
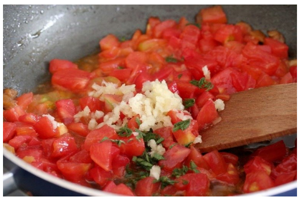
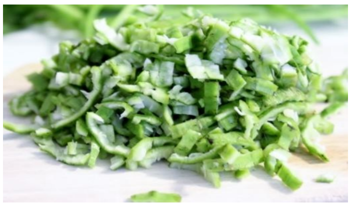
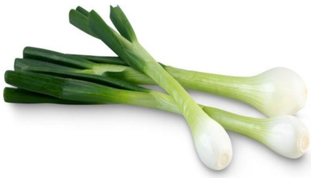
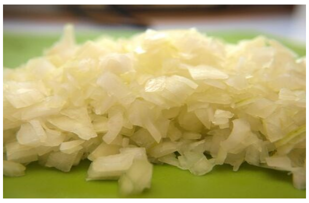
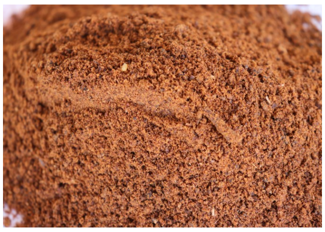
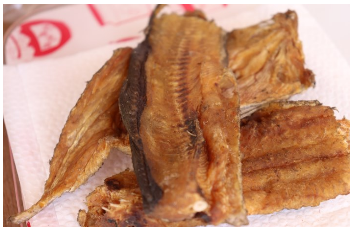
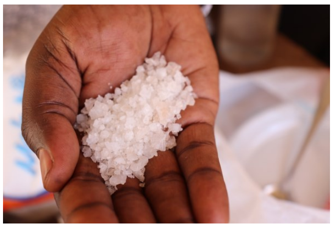
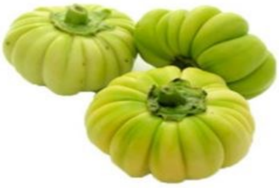
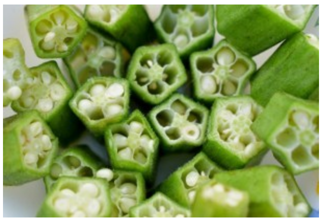
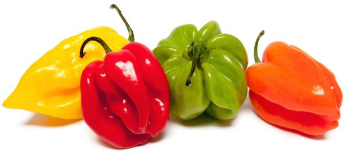
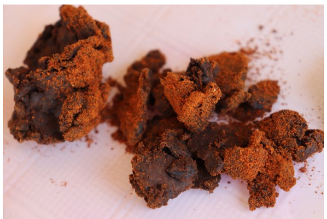
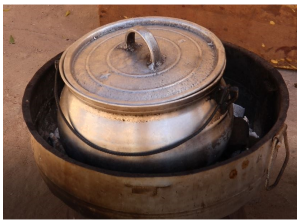

d. ETAPE 3 : LA FINITION :

- Couvrir et laisser cuire jusqu'à la cuisson finale.

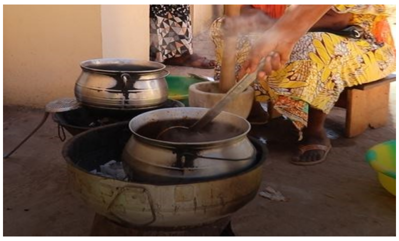
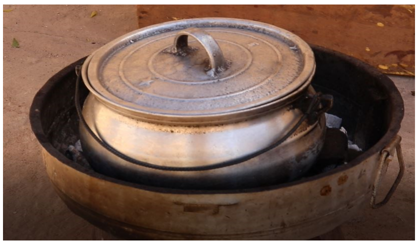
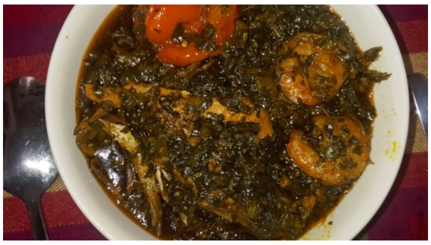

- «SAKA SAKA », à base de poisson frais : le mode de préparation est relativement identique à celle à base de viande. L'unique différente : les poissons doivent être faits bouillir avant de les ajouter à la sauce vers la fin de la cuisson.

4. **RECETTE:** « SAUCE GOMBO FRAIS », Avec la viande de bœuf.

a. INGRÉDIENTS :

- ½ kg de viande ou d'une quantité;
- 4 grandes cuillérées d'huile d'arachide;
- 1 kg de gombo frais;
- Tomate fraiche, Oignon frais;
- 100g de Pate de tomate, soit 1 grande cuillérée;
- 30g de poudre de Soumbala soit 2 petites cuillérées,
- 40g Oignon;
- Poudre de poisson séché soit « wouloudjèguè »;
- Poivre et lauriers à volonté, une cuillérée de céleri et une gousse d'ail;
- Légumes: piments, aubergines africains;
- 1 petite cuillère de sel d'un sachet de 25F.

b. ETAPE 1 : LES PRÉPARATIFS :

- **Enlever la graisse de la viande, couper les légumes en morceaux, préparer les épices.**

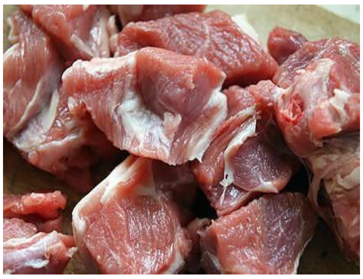
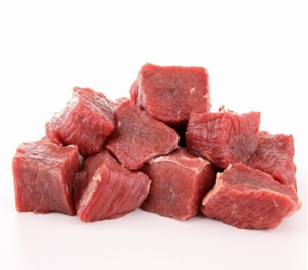
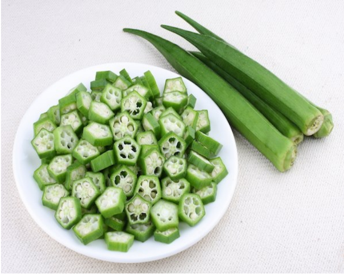
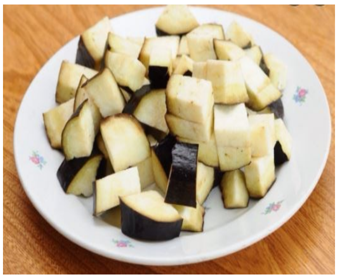
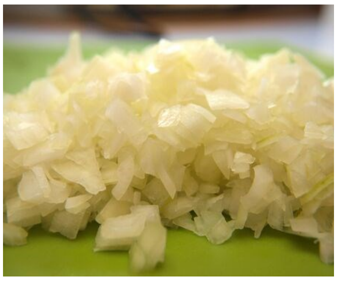
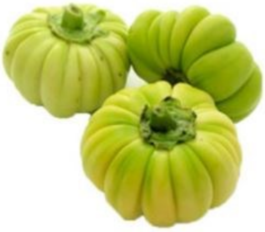
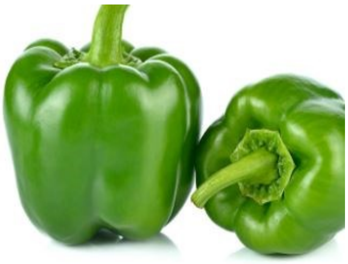
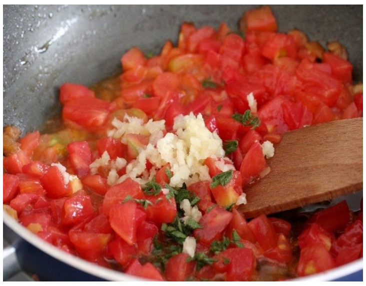
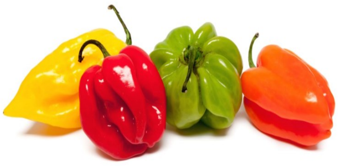
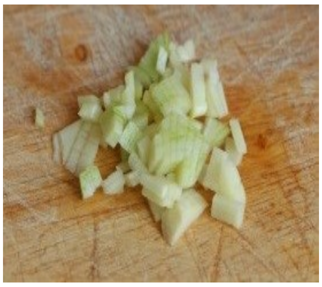
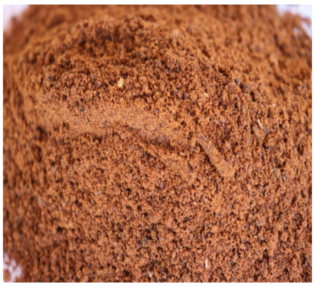
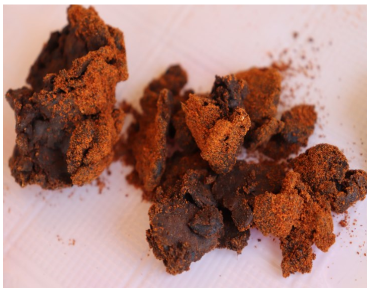

C. ETAPE 2 : LA CUISSON:

- **Mettre l'huile et la viande, et remuer pour 10mn. Puis ajouter des tomates et oignons frais, de la pate de tomate. Laisser cuire encore 5mn, puis ajouter les gombos, les aubergines, le piment frais, et l'assaisonner avec des épices comme la poudre de soumbala, d'oignon frit, de poison séché et le sel. Ajouter de l'eau et laisser cuire jusqu'à la fin.**

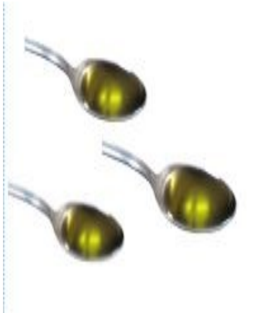
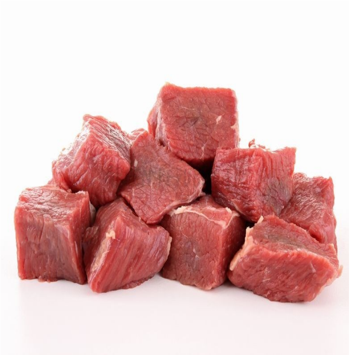
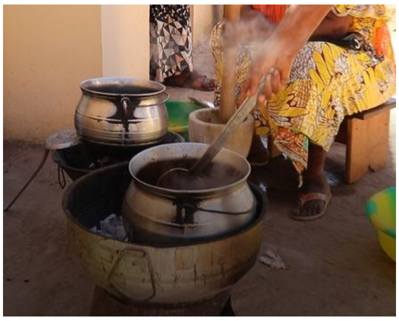
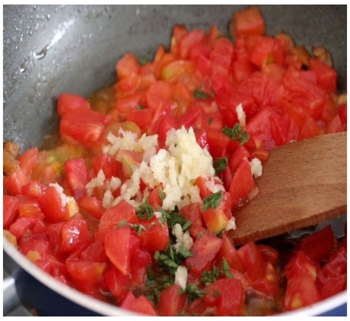
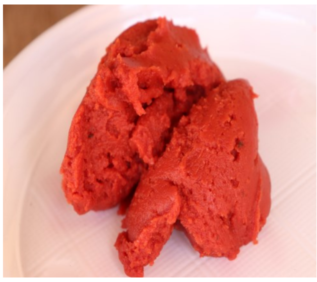
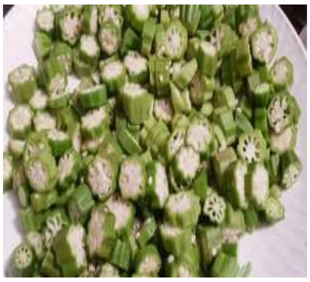
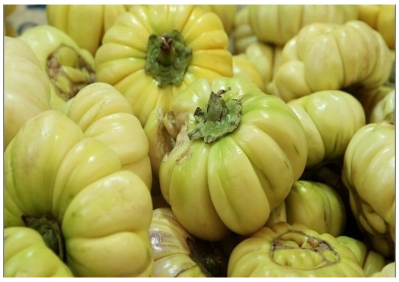
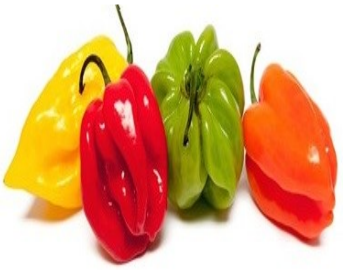
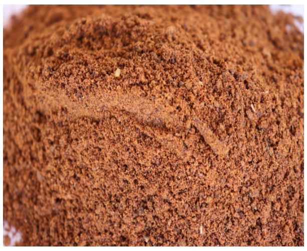
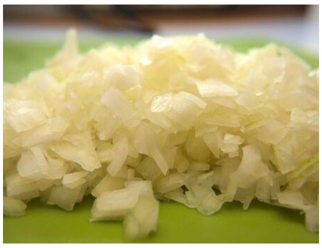
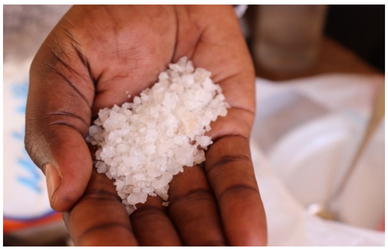
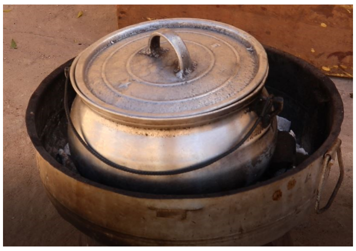

d. ETAPE 3 : LA FINITION :

- **Ajouter l'ail pilé, de poudre de poivre**

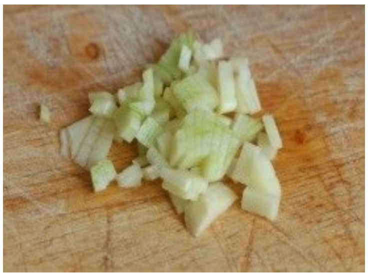
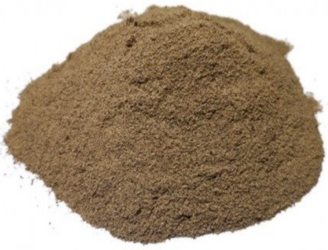
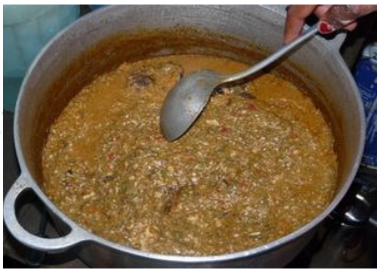
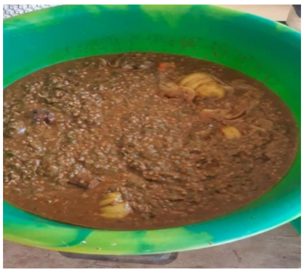

- « **SAUCE GOMBO** » **à base de poisson fumé : le mode de préparation est relativement identique à celle à base de viande. La différence : les poissons fumés doivent être ajoutés à la sauce au cours de la cuisson en même temps que les légumes frais.**

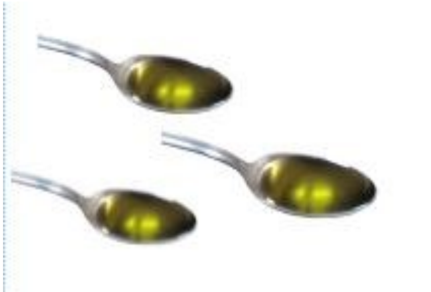
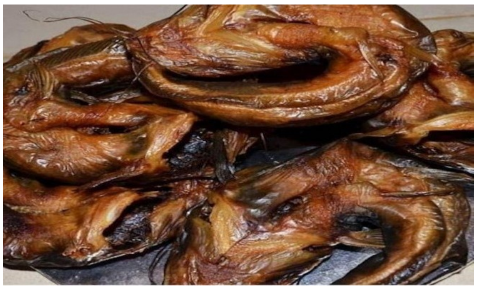
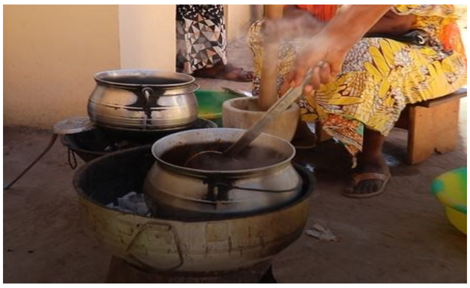
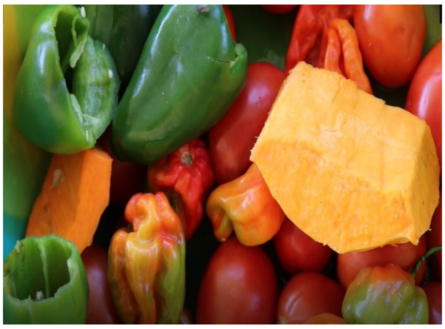
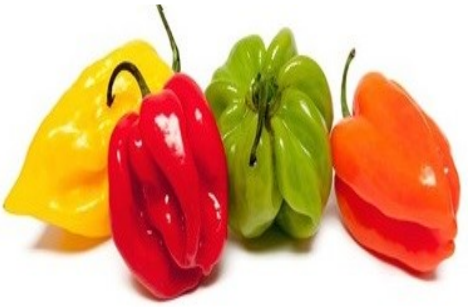
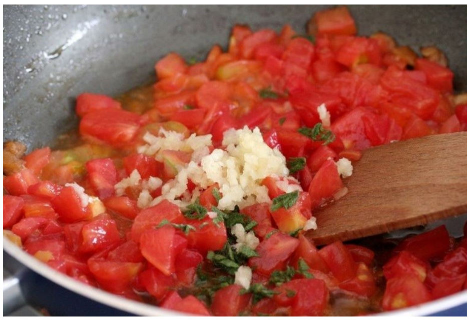
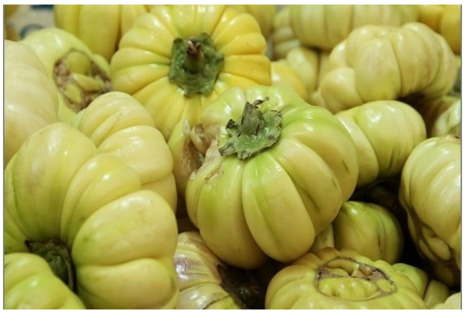
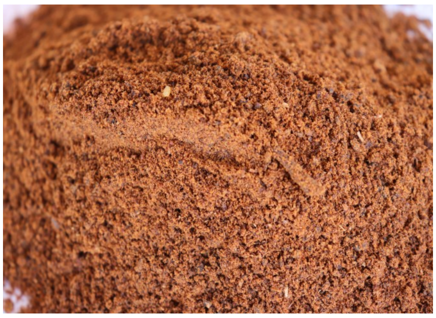
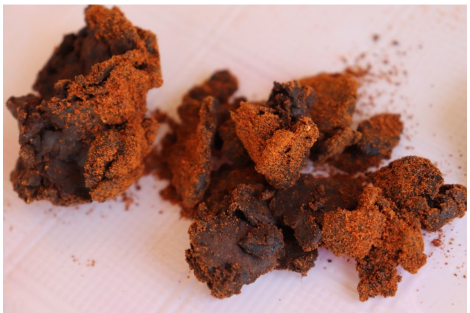
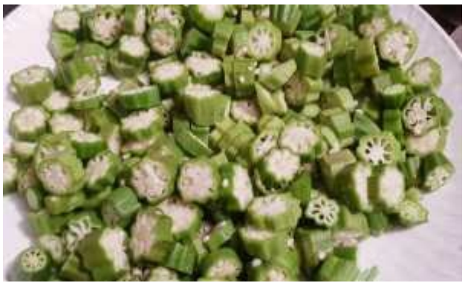
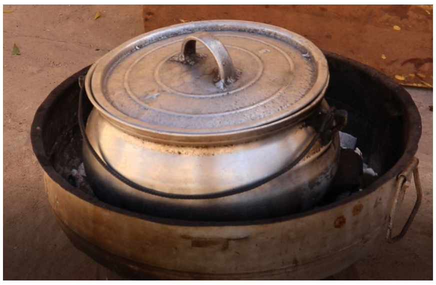
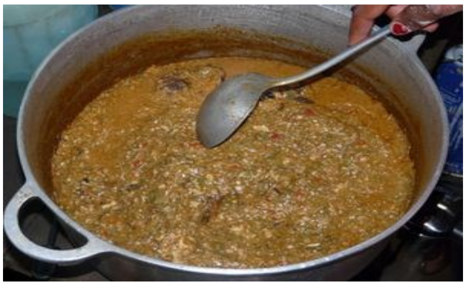

5. RECETTE: «SAUCE FAKOYE - FEUILLE NOIRE », Avec la viande de bœuf, pour 6 personnes

a. INGRÉDIENTS :

- ½ kg de viande de bœuf ou une quantité;
- 3 sachets de 100F Poudre de Fakoye (400g);
- 3 tomates fraiches (250g), Oignon Frais, si nécessaire;
- 2 grandes cuillérées de poudre de Soumbala;
- 4 grandes cuillérées d'huile d'arachide 100g;
- 2 grandes cuillérées de Tikani 5g yirifarani 10g Mafedjai 5g Ganifi;
- 2 sachets de 25 F de Tamaroni (50g), Une poignée Fleurboulouni (5g);
- Légumes : piments et aubergines locales, 200g;
- 1 petite cuillère de sel d'un sachet de 25F

. b. ETAPE 1 : LES PRÉPARATIFS :

- **Enlever la graisse de la viande, couper les légumes en morceaux, préparer les épices.**

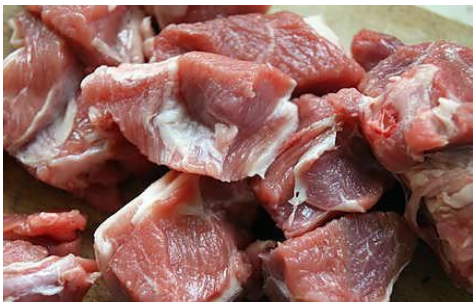
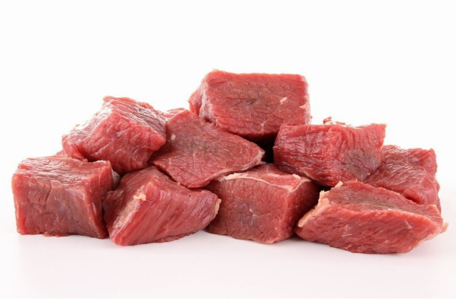
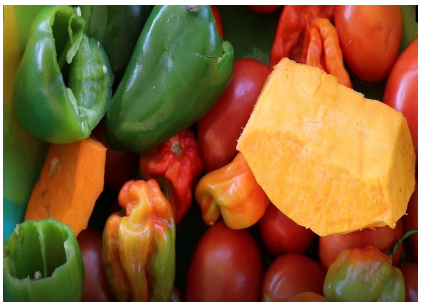
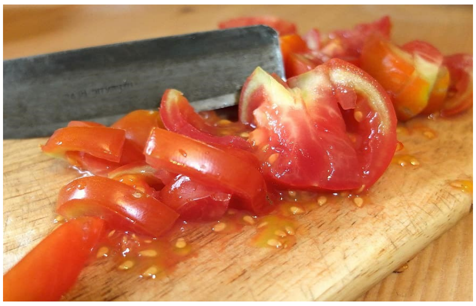
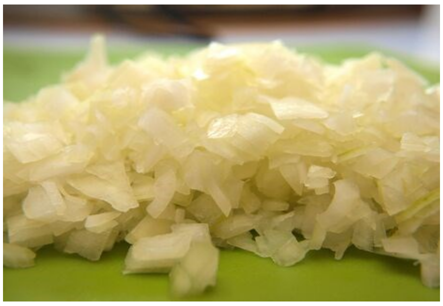
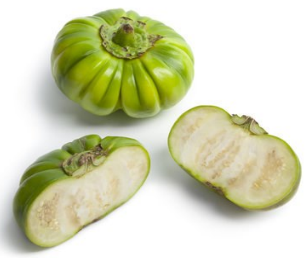
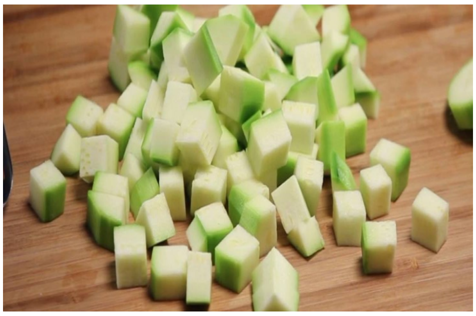
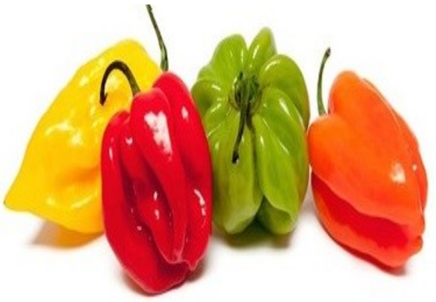
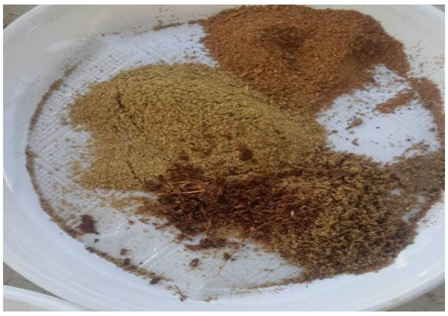

C. ETAPE 2 : LA CUISSON:

- **Mettre l'huile et la viande, et remuer après 10mn. Ajouter les légumes et les remuer. Ajouter de l'eau. Ensuite la poudre de Fakoye malaxée et laisser cuire pour 20mn, puis assaisonner avec des épices.**

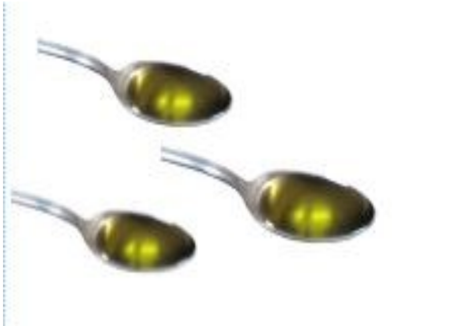
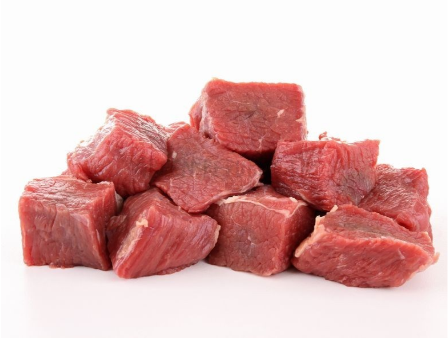
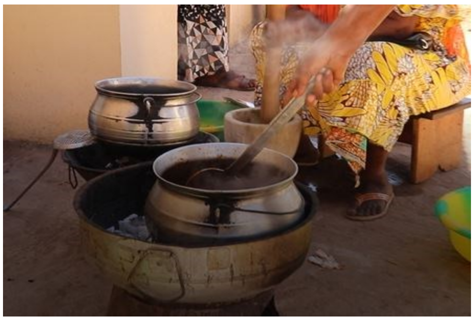
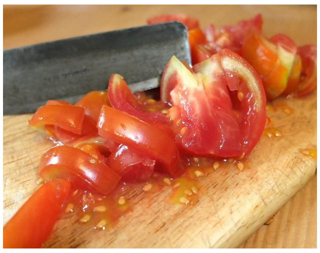
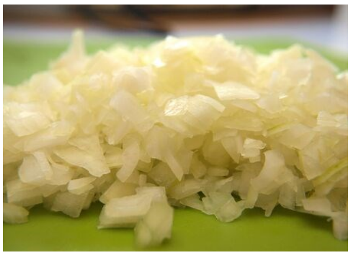
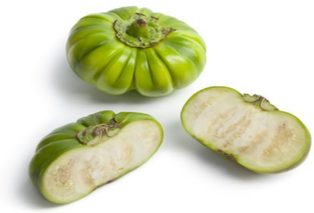
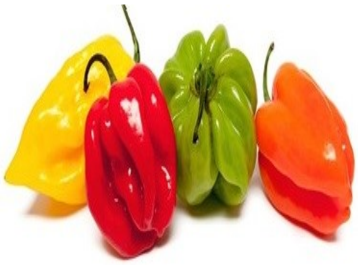
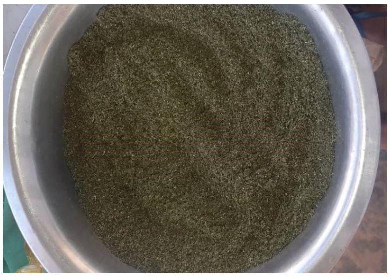
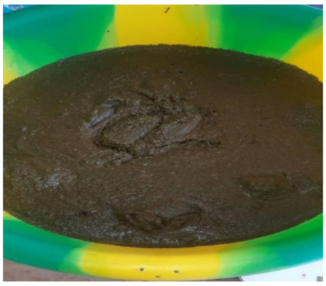
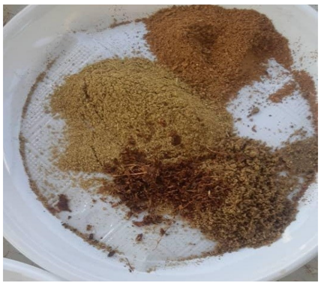
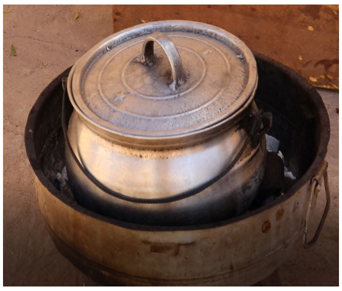

d. ETAPE 3 : LA FINITION :

- **Après une cuisson raisonnable, server la sauce.**

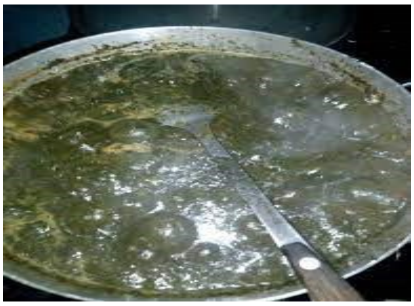
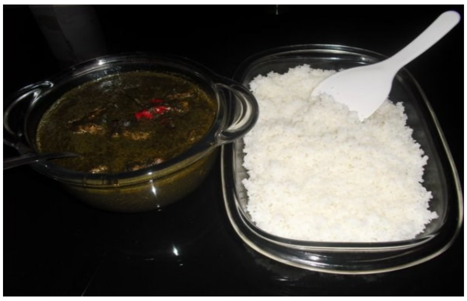
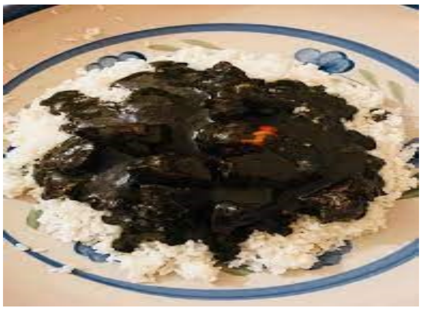

- **« SAUCE FAKOYE » à base de poisson fumé : le mode de préparation relativement est identique à celle à base de viande. L'unique différence : les poissons fumés doivent être ajoutés à la sauce au cours de la cuisson en même temps que les épices.**

6. **RECETTE:** «Zamè », Avec poisson frais, pour 6 personnes

a. INGRÉDIENTS :

- ½ kg d'oignons de petite taille;
- 1 kg de poisson ou une quantité ;
- Légumes : Choux, aubergine locale, poivrons, piments;
- Poisson séché, Yeti, si nécessaire;
- 5 cuillérées d'huile d'arachide;
- **Les épices I** : 1 petites cuillérées du sel, 2 gousses d'ail ;
- **Les épices II:** des feuilles de céleri haché, du persil haché, de feuilles d'oignon haché, de poivre, du fèfè, de feuilles de laurier;
- 1 petite cuillère du sel d'un sachet de 25F

b. ETAPE 1 : LES PRÉPARATIFS :

- **Apprêter les poissons, les épices en 2 groupes (I et II)**

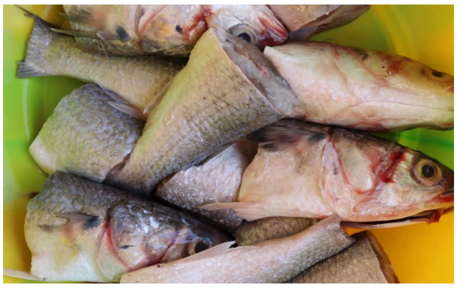
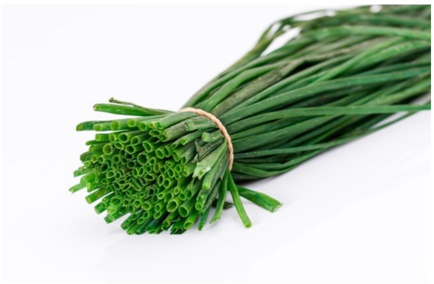
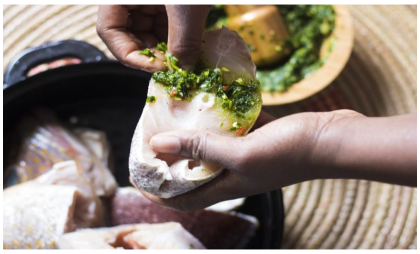
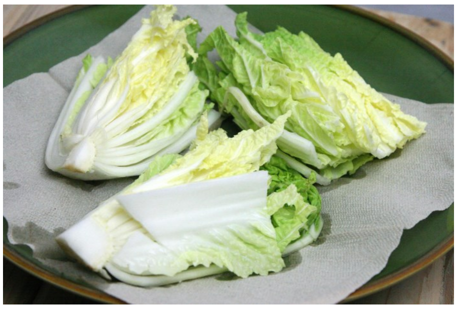
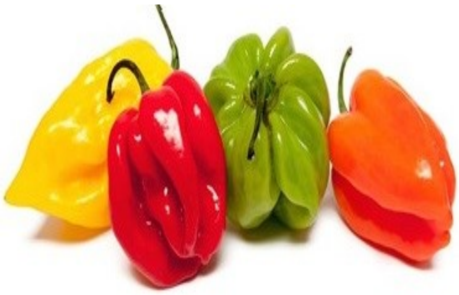
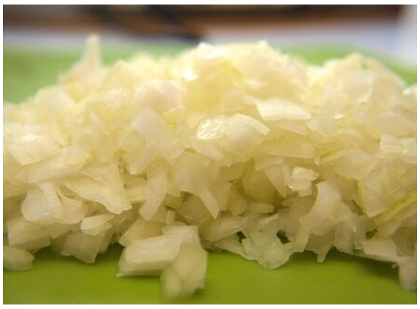
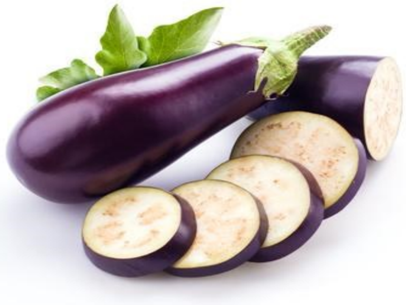
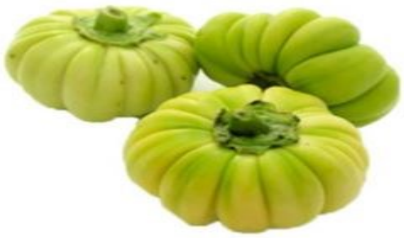
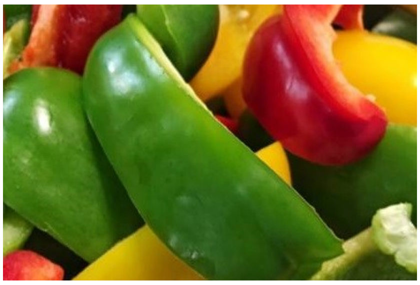

C. ETAPE 2 : LA CUISSON:

- **Mettre les oignons hachés, yeti si nécessaire et les poissons séchés, une fois l'huile chaude, y ajouter de l'eau. Ensuite ajouter une grande cuillérée du mélange des épices , une petite cuillerée de sel, les légumes (Choux, aubergines, carottes, poivrons, piments) ainsi que les morceaux de poisson préalablement assaisonnés avec du persil, l'ail. Après la friture de poissons et ingrédients indiqués, rajouter de l'eau, puis le reste des mélanges d'épices et du sel si nécessaire.**

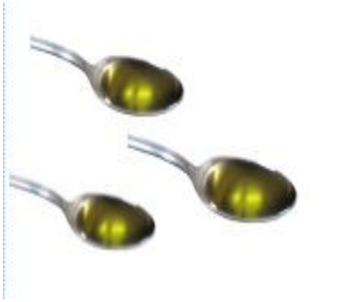
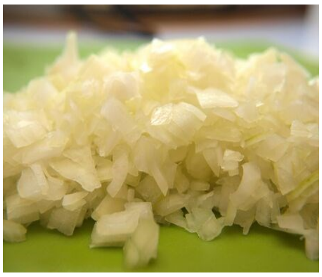
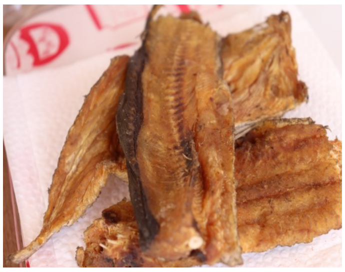
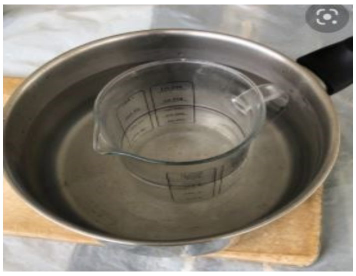
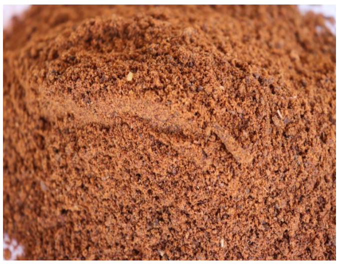
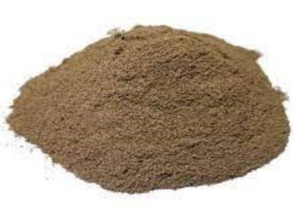
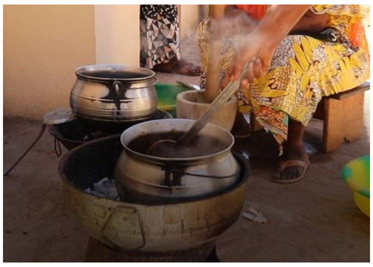
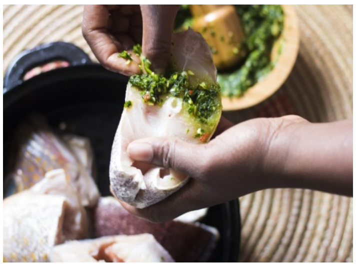
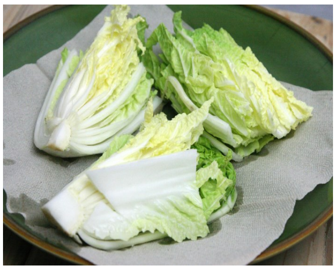
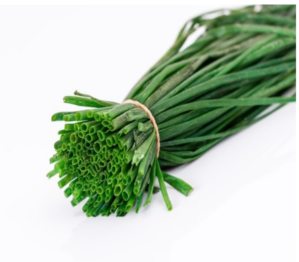
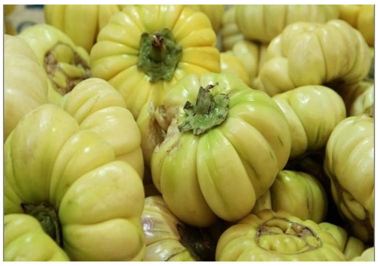
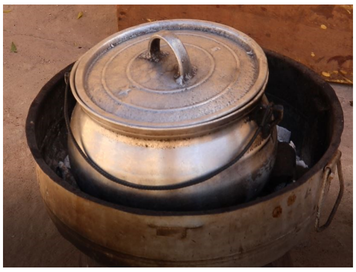

d. ETAPE 3 : LA FINITION :

- **Préparer le riz et ajouter la pate de tomate à la fin. les légumes une fois souples, server le Zamè avec les légumes et les poisson.**

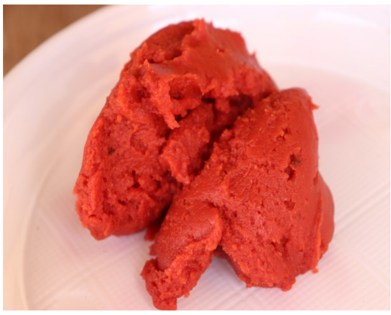
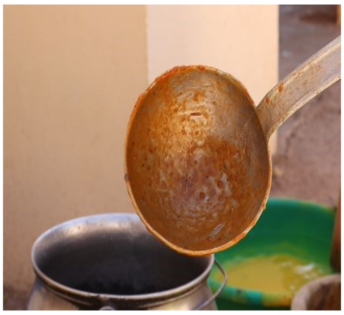
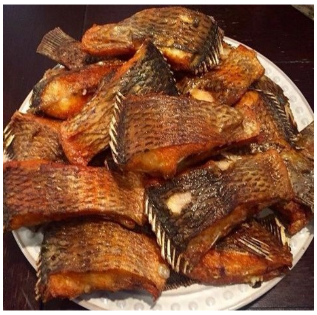
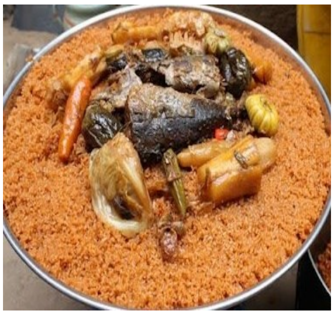

7.[18] RECETTE: « SAUCE YASSA», Avec la viande de bœuf, pour 6 personnes.

a. INGRÉDIENTS :

- ½ kg de viande de bœuf; Oignon 2 kg;
- 2 tomates fraîches;
- Légumes : Chou pomme, aubergine, carotte, piments et d'autres légumes;
- 5 cuillérées d'huile d'arachide
- Epices : : gousses de l'ail, persil pilé, céleri pilé, poudre de poivre, laurier, moutarde, vinaigre; 1 petite cuillère du sel dans un sachet de 25F.

b. ETAPE 1 : LES PRÉPARATIFS :

- **Enlever d'abord les graisses de la viande, puis apprêter les oignons, les légumes et les épices en deux groupes: 1 et 2**

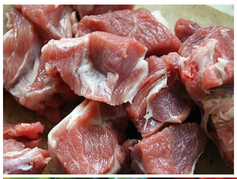
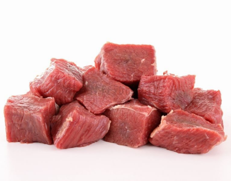
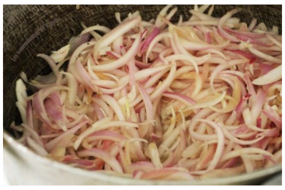
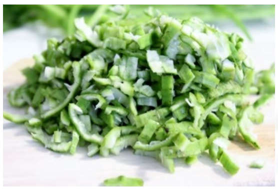
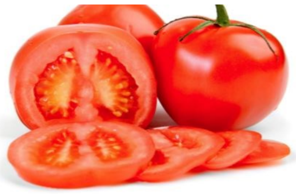
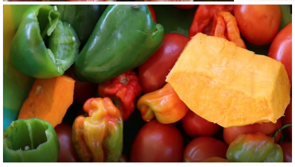
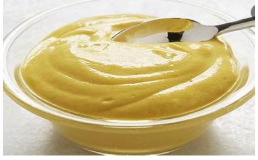
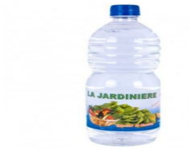

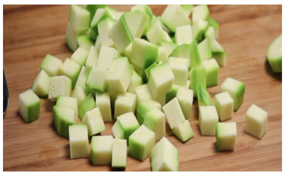
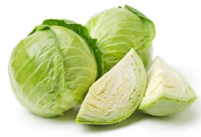

C. ETAPE 2 : LA CUISSON:

- **Griller la viande (poisson ou poulet). Mettre l'arachide dans la marmite et ajouter les oignons et laisser cuire. Ensuite Ajouter tous les autres légumes et les épices du groupe I, l'eau.**

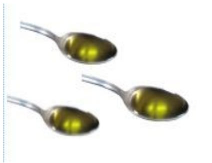
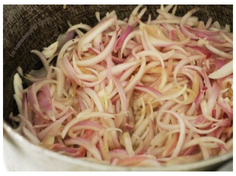
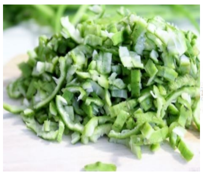
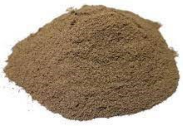
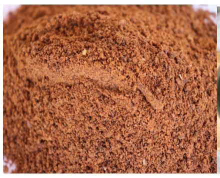
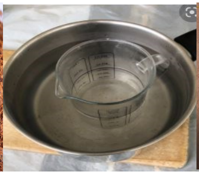
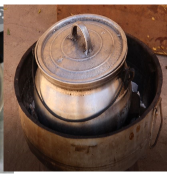

d. ETAPE 3 : LA FINITION :

- **Ajouter à la preparation les épices du groupeII. Une fois les légumes deviennent souples, server la viande (poisson ou poulet) avec sauce.**

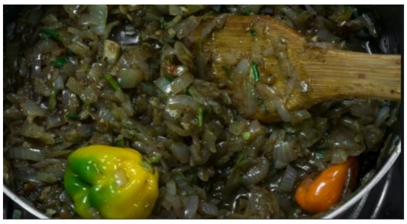
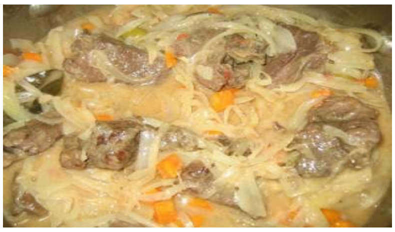
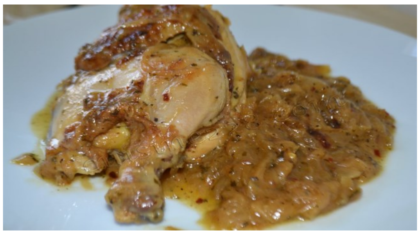

Supplement: Nutrition booklet [file NIHMS2139094-supplement-Nutrition_booklet.pdf]
