# Supplementary material for: A Hybrid Effectiveness-Implementation Trial of the ‘Power To Prevent Diabetes Program’ in Bamako, Mali
Source: Glob Implement Res Appl. Author manuscript; Available in PMC 2026 Feb 5. (PMC12872172; doi:10.1007/s43477-025-00199-x)
Supplement: Weekly food log [file NIHMS2139094-supplement-Weekly_food_log.pdf]

JOURNAL HEBDOMADAIRE DES ALIMENTS

		Lundi	Mardi	Mercredi	Jeudi	Vendredi	Samedi	Dimanche
**PETIT DEJEUNER OU DINER**		#Portions	#Portions	#Portions	#Portions	#Portions	#Portions	#Portions
**Môni (bouillie de mil)**	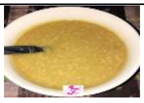							
**Môni + lait caille**	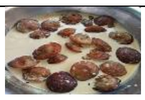							
**Kaba seri (bouillie mais)**	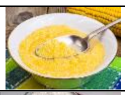							
**Sagnon dègué (crème de mil)**	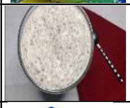							
**Yaourt**	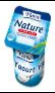							
**Pain avec beurre**	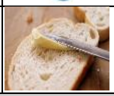							
**Les SNACKS**								
**Galettes de mil (frites)**	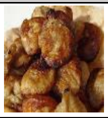							
**Té ou café avec sucre**	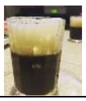							
**Fruit**	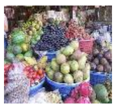							
**Chips**	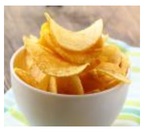							
**Pâtisseries**	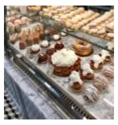							
**Galettes ou beignets**	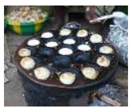							
LES BOISSONS								
**L'Eau**	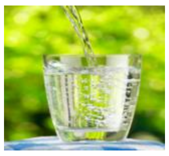							
**Jus de fruits**	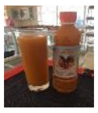							
**Sucrerie (soda)**	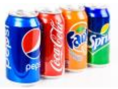							
**Bière-petit**	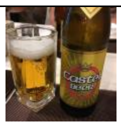							

		Lundi	Mardi	Mercredi	Jeudi	Vendredi	Samedi	Dimanche
**Déjeuner ou Dîner**		#Portions	#Portions	#Portions	#Portions	#Portions	#Portions	#Portions
Les Sauces								
**Sauce oignons (Djabadiij) -**	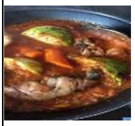							
**Sauce arachide (tigadègué)**	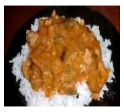							
**Sauces feuilles des haricots (N'ngnougou)**	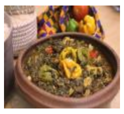							
**Sauce feuilles noires (fakoye)**	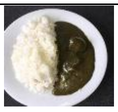							
**Sauce gombo (ghankenena)**	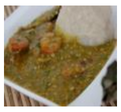							
Additions aux sauces								
**Viande**	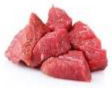							
**Poisson frais**	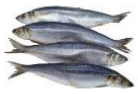							
**Poisson séché**	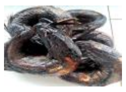							
**Poulet**	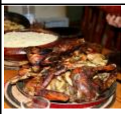							
Les Féculents								
**Riz blanc**	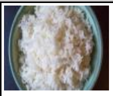							
**Riz au gras (zamè)**	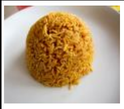							
**Attiéké**	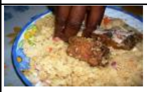							
**Fonio**	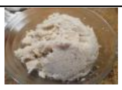							
**Couscous de mil/tô**	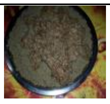							
**Ragout d'igname**	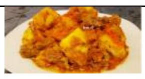							
**Pommes de terre - Frites**	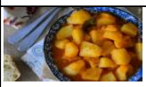							
**Pain**	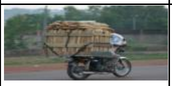							

**Haricots**	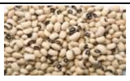							
Les Spéciales								
**Poulet braisé**	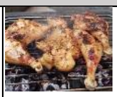							
**Poisson braisé**	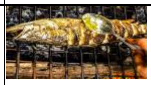							
**Brochettes**	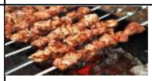							
**Mouton au four**	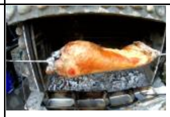							
